# A Cationic Supramolecule With Potent Antifungal Activity, Single‐Species Selectivity, and Strong Synergy With Echinocandins

**DOI:** 10.1002/advs.75480

**Published:** 2026-04-29

**Authors:** Tianjiao Dai, Adrielle Xianwen Chen, Eve Wai Ling Chow, Li Mei Pang, Jianguo Li, Chandra Shekhar Verma, Stefan H. Oehlers, Yue Wang, Ning Li

**Affiliations:** ^1^ A*STAR Infectious Diseases Labs (A*STAR IDL) Agency for Science Technology and Research (A*STAR) Singapore Singapore; ^2^ Bioinformatics Institute (BII) Agency for Science Technology and Research (A*STAR) Singapore Singapore; ^3^ Singapore Eye Research Institute (SERI) The Academia Singapore Singapore; ^4^ Department of Biological Sciences National University of Singapore Singapore Singapore; ^5^ School of Biological Sciences Nanyang Technological University Singapore Singapore; ^6^ School of Chemistry Chemical Engineering and Biotechnology 21 Nanyang Link Nanyang Technological University Singapore Singapore

**Keywords:** antifungal agents, biological activity, peptidomimetics, polycations, supramolecular chemistry

## Abstract

Claiming 3.8 million lives annually, fungal pathogens represent a major health threat, where *Candida* species are the leading cause with >40% mortality rates. Despite rising prevalence of life‐threatening fungal species/strains, progress in developing new antifungal agents remains limited. Here, we report a new synthetic membrane‐active cationic supramolecule **Gua‐SMACS‐16** that exhibits high antifungal potency and selectivity for *Candida tropicalis* (MIC = 0.4‐0.8 µM) without affecting other phylogenetically close species in the *Candida* genus, non‐*Candida* fungal, or common opportunistic bacterial species in the human microbiome. **Gua‐SMACS‐16** exerts its activity by disrupting the cytoplasmic membrane, while its *C. tropicalis* selectivity is attributed to the species’ low cell wall β‐glucan level, which fails to block the supramolecule entry. It also exhibits minimal toxicity to mammalian cells and zebrafish embryos, suggesting a high clinical translation potential. Moreover, an ultra‐strong synergy was observed between **Gua‐SMACS‐16** and caspofungin, a clinical antifungal drug inhibiting 1,3‐β‐glucan synthase, reducing their MICs by orders of magnitude against all tested *Candida* species including the intractable *C. auris*. Together, this work highlights the importance of cell wall glucan for membrane‐active antifungal amphiphiles, provides a novel design principle for species‐specific antifungal agents, and uncovers a new approach to developing synergizers for combination antifungal therapy.

## Graphical abstract

We introduce a new membrane‐active antifungal design, **Gua‐SMACS‐16**, that exhibits high potency and single‐species selectivity for *C. tropicalis* while sparing other close species in the *Candida* genus. Moreover, when combining with caspofungin, a clinical antifungal drug inhibiting 1,3‐β‐glucan synthase, they exhibit ultra‐strong synergy across a panel of important fungal pathogens, reducing the required effective doses by orders of magnitude.

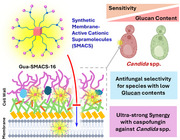

## 1 Introduction

In 2022, the World Health Organization (WHO) published its first‐ever report prioritizing fungal pathogens based on the unmet R&D needs and their serious threat to global public health. *Candida* species are the most common cause of hospital‐acquired fungal infections, with systemic complications resulting in mortality rates exceeding 40%, even with antifungal interventions in place [1]. While *C. albicans* has historically been the predominant cause of fungal infections [2], there has been a notable rise in infections caused by non‐*albicans* species, such as *C. tropicalis*, *C. glabrata* (*Nakaseomyces glabratus*), and *C. auris* in recent years [3]. For instance, in Southeast Asia and South America, *C. tropicalis* has overtaken other *Candida*, becoming the first or second most common cause of bloodstream infection [4]. Fungi are eukaryotic microorganisms sharing most biochemical pathways with humans, making antifungal development particularly challenging [5]. After decades of R&D, only three major classes of antifungal drugs are currently available in clinical settings [6], all targeting fungus‐specific cell surface components: (i) azoles, which inhibit biosynthesis of ergosterol, a crucial steroid present in the cytoplasmic membrane [7]; (ii) polyenes, which bind to and/or extract ergosterol from the membrane [8]; and (iii) echinocandins, which block the synthesis of 1,3‐β‐glucan in the fungal cell wall [9]. Although widely prescribed, these drugs suffer undesirable drug‐drug interactions, high toxicity, and the increasing occurrence of resistance, calling for innovation in new antifungal development, especially designs with novel mechanisms of action (MoA) [10, 11].

In nature, antifungal peptides (AFPs) protect all domains of life against fungal infection, primarily by physical disruption of the fungal cytoplasmic membrane [12]. However, translating these AFPs into drugs is hampered by high production costs and poor in vivo stability due to protease degradation [13]. Such a gap has driven the development of synthetic AFP mimetics with balanced cationic and lipophilic moieties, designed to retain the antifungal potency while overcoming the limitations [14]. Significant efforts have focused on elucidating the membrane‐active antifungal mechanisms [15], developing novel molecular scaffolds [16, 17], identifying intracellular targets [18], understanding species‐specific susceptibility [19], addressing serum sensitivity [20], and optimizing toxicity‐selectivity trade‐offs [21]. The rapid advancement of artificial intelligence (AI) has further equipped researchers with powerful tools to accelerate the discovery of new AFPs and mimetics [22, 23]. Despite considerable progress [24], many important questions remain unanswered or inadequately addressed. For example, what role does the fungal cell wall play in the action of AFP mimetics? Surrounding the cytoplasmic membrane, the dynamic, semi‐rigid fungal cell wall serves as the first point‐of‐contact between the fungus and its environment [25]. The membrane‐active AFP mimetics must diffuse across the polysaccharide layers in the cell wall before reaching the membrane to exert their fungicidal effects. Consequently, the chemically versatile fungal cell wall could be an exploitable target for developing new AFP mimetics, which, to our surprise, remains largely overlooked in the literature [26].

**FIGURE 1:**
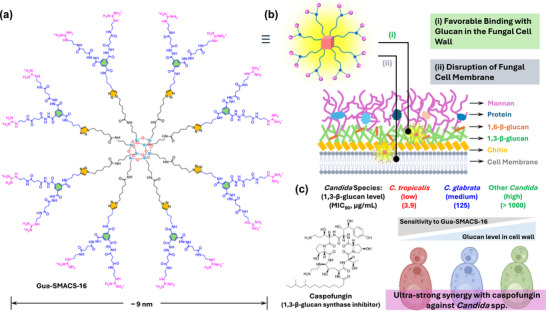
Molecular design of **Gua‐SMACS‐16** and conceptual illustration of its interaction with fungal cell surfaces: (a) Chemical structure; the trifluoroacetate counterions are omitted for clarity. (b) Proposed interaction mechanism with fungal cell wall and cytoplasmic membrane. (c) Correlation of antifungal MIC_90_ values and the glucan levels in the cell walls of different *Candida* species, molecular structure of caspofungin and its ultra‐strong synergy with **Gua‐SMACS‐16** against *Candida* spp.

In this study, we report a synthetic membrane‐active cationic supramolecule, namely **Gua‐SMACS‐16**, that favorably interacts with β‐glucan in the *Candida* cell wall, hence displaying unprecedented high activity and selectivity to *C. tropicalis* (containing lower β‐glucan content, MIC_90_ = 3.9 µg/mL, ≈400 nM) over other phylogenetically related *Candida* (Figure 1), non‐*Candida*, or common opportunistic bacterial species. To our best knowledge, this is the first example of AFP mimetics with both potent efficacy and high selectivity to a single species within the clinically important *Candida* genus, which may be potentially useful for developing narrow‐spectrum antifungals to combat the drug resistance problem or to treat infections without causing microbiome dysbiosis [6]. More interestingly, an ultra‐strong synergy between **Gua‐SMACS‐16** and caspofungin (a clinical antifungal drug that inhibits 1,3‐β‐glucan synthase) was observed, where the combination displays strong, broad‐spectrum efficacy against a panel of *Candida* species prioritized in the 2022 WHO fungal priority pathogen list (FPPL).

## 2 Results and Discussion

### 2.1 Chemical Synthesis and Characterization

A modular approach was employed to synthesize **Gua‐SMACS‐16**. In brief, a Y‐shaped arm functionalized with one alkyne and two Boc‐protected guanidine moieties was first made in seven steps (Scheme S1), which further click‐reacted with an azido‐POSS (polyhedral oligomeric silsesquioxane) to form a giant molecular sphere carrying sixteen Boc‐protected guanidine groups (Scheme S2) [14]. Subsequent deprotection using trifluoroacetic acid (TFA) afforded **Gua‐SMACS‐16** as a pale‐yellow solid. The product was thoroughly characterized by NMR, LC‐MS, GPC, MALDI‐TOF, and HPLC to confirm the molecular structure and chemical purity (see Supporting Information). Guanidinium is preferable over other simpler cationic functionalities (e.g., primary, tertiary, or quaternary amines) for antimicrobial use [27], mainly because: (i) the guanidinium group, with a pKa exceeding 11, remains fully protonated at physiological pH, thereby maintaining a high cationic charge density; (ii) the positive charge on guanidinium cation is delocalized across the three nitrogen atoms, and this resonance renders it greater stability than conventional amine cations, supporting sustained activity; and (iii) the Y‐shaped guanidinium structure enables the formation of bidentate hydrogen bonds with anionic phosphate groups on microbial membrane, promoting a stronger membrane interaction and antimicrobial action [28].

At its most extended conformation, the molecular diameter of **Gua‐SMACS‐16** is estimated to be around 9 nm. Using Griffin's method by classifying the POSS core, guanidinium, and amide groups as hydrophilic, while the aromatic and alkyl moieties as lipophilic groups [29], the hydrophilic‐lipophilic balance (HLB) was calculated to be 10.6, corresponding to the range typical of oil‐in‐water emulsifiers. Notably, the spatial distribution of the hydrophilic and lipophilic domains also plays a critical role in determining physiochemical behavior. Molecular simulation of **Gua‐SMACS‐16** in aqueous solution and at membrane interfaces revealed that the radius of gyration of the hydrophobic moieties is consistently smaller than that of the hydrophilic components (Figure S1). This suggests that the hydrophilic groups are predominantly surface‐exposed, while lipophilic segments are preferentially localized toward the core. As a result, no stable particle size or zeta potential could be experimentally detected at concentrations up to 200 µg/mL in aqueous solution, and no micelle formation was observed either within this range. The aqueous solubility of **Gua‐SMACS‐16** is >50 mg/mL, which readily enables the subsequent microbiological characterization and evaluation.

### 2.2 Potent Antifungal Activity and High Selectivity

**FIGURE 2:**
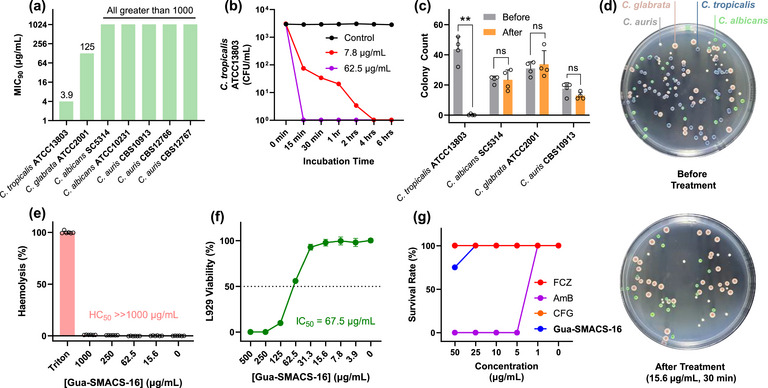
Antifungal activity and selectivity of **Gua‐SMACS‐16**. (a) Minimum inhibitory concentration, MIC_90_, against different *Candida* species. (b) Fungicidal kinetics against *C. tropicalis* ATCC13803. (c) Viable CFU quantification, and (d) photographs of representative CHROMagar plates of a co‐culture model to show selectivity towards *C. tropicalis*. (e) Negligible haemolytic activity against red blood cells of C57BL/6 mice. (f) Low in vitro cytotoxicity against L929 mouse fibroblast cells. (g) Acute toxicity of **Gua‐SMACS‐16** and comparator antifungal drugs including fluconazole (FCZ), Amphotericin B (AmB), and caspofungin (CFG) against zebrafish embryos (n = 20).

A panel of standard laboratory strains of *Candida* spp., including *C. albicans* SC5314, *C. albicans* ATCC10231, *C. glabrata* ATCC2001, *C. tropicalis* ATCC13803, *C. auris* CBS10913, *C. auris* CBS12766, and *C. auris* CBS12767, were screened using a method adapted from the Clinical Laboratory Standards Institute (CLSI) microdilution protocol to determine the 90% minimum inhibitory concentration (MIC_90_). Surprisingly, strong antifungal activity was only observed against *C. tropicalis* ATCC13803 (MIC_90_ = 3.9 µg/mL, 400 nM), in sharp contrast with other *Candida* species where the MIC_90_ values were 125 µg/mL for *C. glabrata* ATCC2001 and >1000 µg/mL for all others (Figure 2a). To verify this unexpected selectivity, we performed additional testing using a panel of clinical isolates of *Candida* spp., including all the species mentioned above, as well as *C. dubliniensis*, *C. guilliermondii*, *C. krusei*, and *C. parapsilosis* (Table S1). The MIC_90_ values were all equal to or greater than 125 µg/mL, except for that against *C. tropicalis*, where they were mostly in the 3.9–7.8 µg/mL range. In addition, we further assessed the antimicrobial activity of **Gua‐SMACS‐16** against *A. fumigatus* A1160 (another clinically important fungal pathogen designated as Critical Priority by WHO) together with a panel of opportunistic/commensal bacterial species present in the human microbiome, including *E. coli*, *S. aureus*, MRSA, *K. pneumoniae*, *A. baumannii*, *P. aeruginosa*, *E. faecium*, and *E. faecalis*. In all cases, the MIC_90_ values were greater than or eqeual to 125 µg/mL (Figure S2), corroborating the high *C. tropicalis* selectivity of **Gua‐SMACS‐16**.

To determine whether the inhibitory effect against *C. tropicalis* is fungicidal or fungistatic, the suspension from each clear well of the above MIC assay for *C. tropicalis* ATCC13803 was plated onto YPD agar and incubated at 30°C for 48 h. The minimum fungicidal concentration (MFC), defined as the lowest concentration that eradicates the inoculum (≈5 × 10^2^ CFU/mL), was found to be 7.8 µg/mL (2× MIC_90_), suggesting that **Gua‐SMACS‐16** is a fungicide. The fungicidal kinetics were established by quantifying the number of surviving cells at different incubation time points (Figure 2b). At 7.8 µg/mL, **Gua‐SMACS‐16** eliminated the inoculum (≈5 × 10^3^ CFU/mL) within 4 h. At 62.5 µg/mL, the elimination occurred within 15 min, suggesting a rapid fungicidal action and a possible MoA of physical attack on the cell surface [30]. Such a physical mechanism could be further implied from the slightly decreased activity in the presence of high ionic strength in the culture media (Figure S3), a phenomenon observed for many membrane‐disrupting AFPs in the literature [31]. Furthermore, the MIC_90_ increased from 3.9 to 7.8 µg/mL in the presence of 10% Fetal Bovine Serum (FBS), and further to 15.6 µg/mL with 20% FBS, consistent with the serum sequestration phenomena well documented in the literature (Figure S4a) [20]. In parallel, the antifungal activity was also assessed in the presence of Bovine Serum Albumin (BSA, a major protein component of FBS) at concentrations up to 250 µg/mL. Interestingly, the MIC_90_ remained unchanged or was slightly reduced (Figure S4b), suggesting that the protein does not significantly affect the activity of **Gua‐SMACS‐16** against *C. tropicalis* and the reduced activity in FBS is likely attributable to other serum components. The fungicidal nature of **Gua‐SMAS‐16** further allowed verification of its selectivity using an in vitro co‐culture method. A mixed culture of four representative *Candida* species, including *C. albicans* SC5314, *C. glabrata* ATCC2001, *C. auris* CBS10913, and *C. tropicalis* ATCC13803, were incubated with 15.6 µg/mL **Gua‐SMACS‐16** (2× MFC) for 30 min before plating onto CHROMagar. As shown in Figure 2c,d, only *C. tropicalis* cells were eliminated, while the others remained largely unaffected.

Within the *Candida* genus, *C. tropicalis* is a highly efficient biofilm producer, surpassing other clinically important species, such as *C. albicans*, in its ability to form the complex community of cells encased in a self‐produced extracellular matrix (ECM) [32]. This ECM provides a protective environment for the encased fungal cells, making them far more resistant to antifungal treatments and host immune attack compared to their planktonic counterparts [33]. The antibiofilm performance of **Gua‐SMACS‐16** was investigated using *C. tropicalis* ATCC13803 as a model organism [14]. The biofilm was grown to maturity before the spent media was replaced with fresh media containing **Gua‐SMACS‐16** at different concentrations [34]. After 24 h of treatment and gentle washing to remove unattached cells, the remaining biofilm was quantified by crystal violet (CV) staining. As shown in Figure S5, **Gua‐SMACS‐16** exhibited a clear dose‐dependent effect against the mature biofilms [35]. For instance, 125 µg/mL of **Gua‐SMACS‐16** reduced the biofilm by approximately 77%, comparable to some naturally occurring AFPs, such as HsAFP1 [36], and the reduction was further increased to about 94% with 500 µg/mL of **Gua‐SMACS‐16** in place.

### 2.3 Minimal Haemolysis, Cytotoxicity, or Acute Toxicity

Next, the in vitro haemolytic activity and cytotoxicity of **Gua‐SMACS‐16** were evaluated to assess its selectivity for fungal over mammalian cells. As shown in Figure 2e, no haemolysis of red blood cells from C57BL/6 mice was observed even at the highest tested concentration of 1000 µg/mL (i.e., 250× MIC_90_), indicating that the polycationic supramolecule has much higher selectivity for *C. tropicalis* over red blood cells. For comparison, the commonly prescribed amphiphilic antifungal drug amphotericin B (AmB), which binds to and extracts ergosterol from the fungal cytoplasmic membrane, can barely differentiate between the fungal and red blood cells, showing an MIC_90_ of 1.0 µg/mL against *C. tropicalis* ATCC13803 and an HC_50_ of only 3.0 µg/mL under identical experimental conditions. The in vitro cytotoxicity of **Gua‐SMACS‐16** was evaluated on L929 mouse fibroblast cells using the alamarBlue cell viability assay [14]. The IC_50_, i.e., the minimum concentration required to inhibit 50% of cell viability, was found to be 67.5 µg/mL after 48 h of incubation (Figure 2f), more than 17 times higher than its MIC_90_ against *C. tropicalis* ATCC13803. At the fungicidal concentration of 7.8 µg/mL, approximately 99.5 ± 4.4% of the fibroblast cells remained viable after 48 h, corroborating a low toxicity to mammalian cells.

In addition, a zebrafish toxicity model was employed to evaluate the acute toxicity of **Gua‐SMACS‐16** to embryos at 2 days post‐fertilization [37]. As shown in Figure 2g, 15 out of the 20 (i.e., 75%) embryos survived the 4‐hour treatment with 50 µg/mL of **Gua‐SMACS‐16**. In comparison, the surviving rates were 0% for the AmB treatment group. At a lower concentration of 25 µg/mL, all 20 embryos in the **Gua‐SMACS‐16** group survived, while surviving rate for the comparator drug AmB remained at 0%. A similar trend, i.e., acute toxicity following the order of AmB > **Gua‐SMACS‐16**, was also observed with longer incubation periods up to 24 h (Figure S6). Combined, our results clearly showed that **Gua‐SMACS‐16** displayed similar efficacy to the membrane‐targeting antifungal drug AmB but much lower toxicity, suggesting its high potential for clinical translation [38].

### 2.4 Fungal Membrane Disruption

**FIGURE 3:**
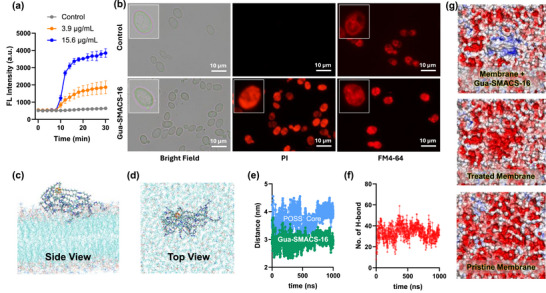
Interaction between **Gua‐SMACS‐16** and fungal cell membranes. (a) Time‐dependent profile of PI fluorescence intensity to monitor the cell membrane permeability upon **Gua‐SMACS‐16** treatment at different concentrations. (b) Bright field, PI and FM4‐64 fluorescence microscopic images of *C. tropicalis* ATCC13803 cells with and without treatment. Final state of a molecular dynamics simulation system containing one **Gua‐SMACS‐16** molecule and a model fungal membrane patch: (c) side view and (d) top view. (e) Simulated distances between the mass center of the POSS core or the entire molecule and center of the lipid bilayer. (f) Number of hydrogen bonds between **Gua‐SMACS‐16** and the model membrane lipids. (g) **Gua‐SMACS‐16** modifies the electrostatic potential of fungal cell membrane surface; red area denotes negative, while blue denotes positive charges.

As most AFPs and peptidomimetics exert antifungal activity through cytoplasmic membrane disruption [12], we hypothesize the same for **Gua‐SMACS‐16**. The propidium iodide (PI) fluorescence assay was firstly performed to monitor the cytoplasmic membrane permeability [14]. PI, a DNA‐binding dye molecule, is normally impermeable to intact lipid bilayer membranes; however, when membranes are compromised, it can readily diffuse into the nucleus, producing strong red fluorescence upon DNA intercalation. As depicted in Figure 3a, treatment of *C. tropicalis* ATCC13803 cells with 3.9 µg/mL (1× MIC_90_) of **Gua‐SMACS‐16** led to a time‐dependent increase in fluorescence intensity, indicating that the polycationic supramolecule can induce structural damage to the fungal membrane. For visual confirmation, microscopic images were taken of *C. tropicalis* ATCC13803 cells with and without **Gua‐SMACS‐16** treatment (Figure 3b). Despite maintaining a similar oval shape, treated cells exhibited abnormal cell surface morphology and altered intracellular content. Strong PI fluorescence signals were observed throughout the cytoplasm and nuclei of all treated cells, further indicating that **Gua‐SMACS‐16** induces structural damage to both the cytoplasmic and nuclear membranes of the fungal target [39]. As an additional evidence, the DiSC_3_(5) fluorescence assay also confirmed that **Gua‐SMACS‐16** induces cytoplasmic membrane depolarization in the *C. tropicalis* ATCC13803 cells (Figure S7). The lipophilic styryl dye FM4‐64 was then used to visualize the intracellular vacuoles of *C. tropicalis* ATCC13803 [40]. Fungal vacuole is a membrane‐bound organelle that plays important roles in multiple physiological processes, including homeostasis, cellular trafficking, and stress tolerance [41]. Our results showed that intracellular vacuoles of untreated *C. tropicalis* ATCC13803 can be clearly labelled with FM4‐64 [42], whereas with **Gua‐SMACS‐16** treatment, the fluorescence signal dispersed throughout the cytoplasm region (Figure 3b), suggesting a significant structural damage to vacuoles and possibly other membrane‐bound organelles [43]. However, it remains unclear at this stage whether the observed intracellular membrane disruption is directly triggered by **Gua‐SMACS‐16** or is a downstream consequence of the cytoplasmic membrane damage.

To gain further understanding on how **Gua‐SMACS‐16** possibly interacts with the fungal membrane, molecular dynamics (MD) simulations were conducted. The simulation system comprises one **Gua‐SMACS‐16** molecule and a model membrane patch consisting of 288 lipids with a POPC:POPE:POPS:ergosterol (ERG) molecular ratio of 4:2:1:2 [44]. As shown in Figure 3c–e, **Gua‐SMACS‐16** can rapidly adsorb onto the membrane patch, possibly driven by electrostatic interactions between the cationic guanidinium groups and the anionic membrane surface. Proximal radial distribution function (pRDF) suggests that the membrane‐bound POSS core has a slightly higher degree of hydration than it does in water (Figure S8). This happens potentially because, upon membrane interaction, the cationic arms bind to the lipid head groups, exposing the POSS core, whereas in the absence of the membrane, the hydrophobic arms mostly wrap around the POSS core. Upon adsorption onto the membrane surface, the distance between **Gua‐SMACS‐16** and the center of the lipid bilayer remains nearly constant throughout the simulation (Figure 3e), indicating a favorable and stable surface binding. Approximately 35–40 hydrogen bonds were formed between **Gua‐SMACS‐16** and the membrane surface in our model, averaging 4.5 per arm for each of the eight Y‐shaped arms (Figure 3f). Moreover, **Gua‐SMACS‐16** seems able to recruit surrounding anionic lipids, resulting in anion‐rich micro‐domains, as shown by the electrostatic potential change of the model membrane surface (Figure 3g). The formation of lipid microdomains may create line tension at the domain edges, affecting membrane fluidity, structure, and consequently membrane and membrane‐bound protein functions. We note that our MD simulation was conducted using a simplified membrane model comprising only four types of lipids and the results are mostly hypothetical. Nevertheless, the findings provide preliminary insights into the molecular interaction details between **Gua‐SMACS‐16** and the fungal cell surface, warranting more comprehensive biophysical investigations.

Further experimental screening was conducted to assess the impact of exogeneous microbial lipids or steroids on the antifungal efficacy of **Gua‐SMACS‐16**. As shown in Figure S9, the MIC_90_ against *C. tropicalis* ATCC13803 remained unchanged in the presence of neutral steroids or zwitterionic lipids, but increased markedly with anionic lipids, highlighting the critical role of electrostatic interactions in governing the membrane disruption and, consequently, antifungal efficacy.

### 2.5 Transcriptomic Response

**FIGURE 4:**
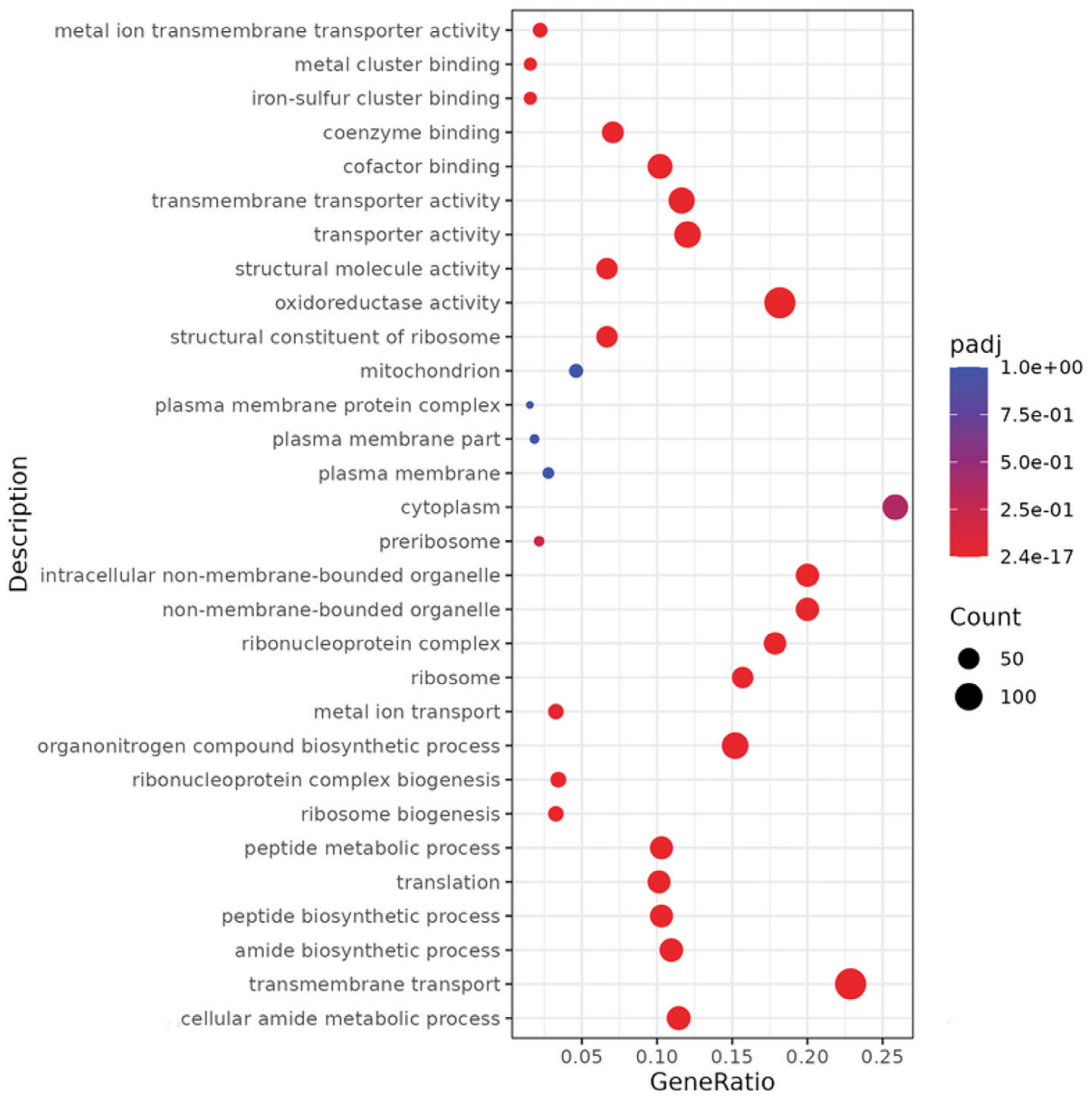
Gene Ontology (GO) enrichment analysis of *C. tropicalis* ATCC13803 cells upon grown for 6 h in the presence of **Gua‐SMACS‐16** at a sub‐MIC concentration. The x‐axis represents ratio of the number of differential genes linked with the GO Term to the total number of differential genes, and the size of a dot represents the number of genes annotated to a specific GO Term. The color from red to blue represents the significant level of the enrichment.

Aiming at a broad, cellular‐level understanding of the antifungal action, we profiled the transcriptomic response of *C. tropicalis* ATCC13803 cells cultured for 6 h with sub‐inhibitory concentrations of **Gua‐SMACS‐16**, where 870 upregulated and 1012 downregulated genes relative to untreated control were identified. From the Gene Ontology (GO) enrichment analysis, the most notable changes were in the categories of transmembrane transport, oxidoreductase activity, as well as multiple cellular components or processes such as organelles, ribosomes, and biosynthesis (Figure 4). With the membrane disruption action described above, it is expected that functions of many membrane proteins, especially transporters, will be affected. The observed change in oxidoreductase activity could be multifactorial, potentially reflecting a cellular defense response to reactive oxygen species (ROS)‐induced stress, or arising from the direct modulation of redox‐related enzyme activity or expression by **Gua‐SMACS‐16**, ultimately resulting in an imbalanced redox state [45]. ROS production is a common mechanism of many AFPs to kill fungal pathogens by damaging cellular components and/or triggering apoptosis [46]. Experimentally, we observed that the MIC_90_ of **Gua‐SMACS‐16** against *C. tropicalis* ATCC13803 increased by 2–4 folds in the presence of the reducing agent sodium ascorbate, suggesting a measurable role of oxidoreductive activity in its antifungal action (Figure S10) [47]. We next sought to assess potential H_2_O_2_ generation using a bioluminescent assay kit. In contrast to the menadione positive control, no detectable H_2_O_2_ was observed when *C. tropicalis* ATCC13803 cells were incubated with 3.9 or 15.6 µg/mL **Gua‐SMACS‐16** (Figure S11). Then DCFH‐DA (i.e., 2'‐7'‐dichlorofluorescin diacetate) was employed as a fluorescence probe to detect ROS in general. Surprisingly, the fluorescence intensity dropped when mixing 15.6 µg/mL **Gua‐SMACS‐16** with the sensitive *C. tropicalis* ATCC13803 or CW0271 strains, while remained unchanged for the resistant CW0366 or *C. albicans* SC5314 strains (Figure S12). In other words, **Gua‐SMACS‐16** seems unable to generate ROS itself for antifungal action but instead induces oxidoreductive stress and inhibits the intrinsic physiological activity of the fungal target, though alternative explanations, such as interference by **Gua‐SMACS‐16** or the altered cellular state with DCFH‐DA's cross‐membrane transportation, hydrolysis, (self‐)oxidation, or photo‐degradation processes, cannot be ruled out [48].

### 2.6 Mechanisms Behind the C. tropicalis Selectivity

**FIGURE 5:**
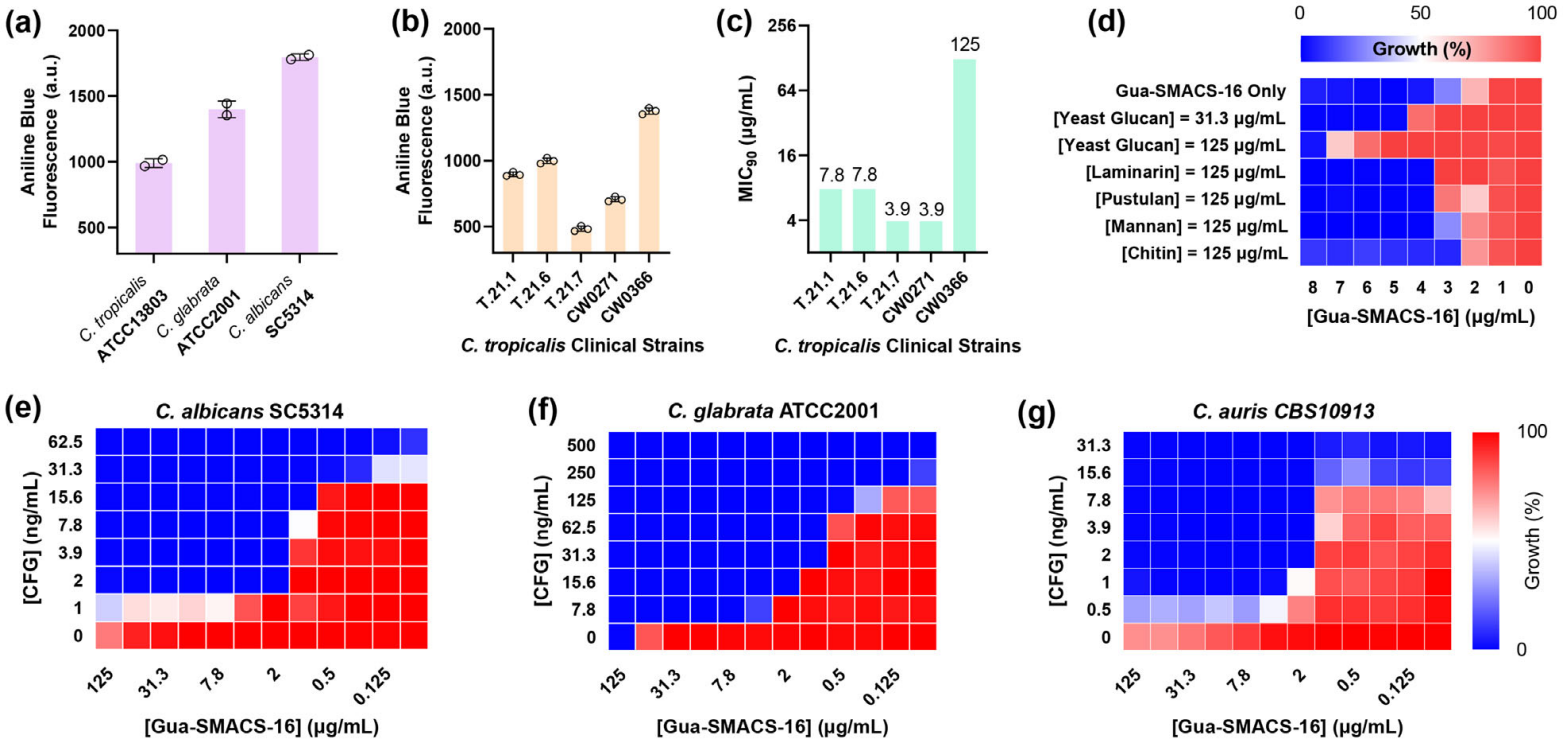
Interaction of **Gua‐SMACS‐16** with fungal cell wall and its ultra synergy with caspofungin. (a) Quantification of glucan levels in different *Candida* species using aniline blue staining. (b) Quantification of glucan levels in different clinical isolates of *C. tropicalis* and (c) their respective MIC_90_ values. (d) Change of **Gua‐SMACS‐16**’s MIC_90_ against *C. tropicalis* ATCC13803 in the presence of different cell wall polysaccharides. Checkerboard assay to show the synergy between **Gua‐SMACS‐16** and caspofungin against (e) *C. albicans* SC5314, (f) *C. glabrata* ATCC2001, and (g) *C. auris* CBS10913.

Returning to the antifungal selectivity, we sought to understand why *C. tropicalis*, but not other phylogenetically close *Candida* species, is sensitive to **Gua‐SMACS‐16**. Given the giant molecular size, we initially suspected that the cell wall of *C. tropicalis* might be more porous than that of others, allowing for easier diffusion of **Gua‐SMACS‐16**. However, this hypothesis was not supported by our experimental measurements of *Candida* cell wall porosity (Figure S13) [49]. We then examined the abundance of different cell wall polysaccharides and found a correlation between the MIC_90_ value and β‐glucan level. For example, *C. tropicalis* ATCC13803 contains the lowest glucan level (previously reported [50] and verified here by aniline blue staining [51]), followed by *C. glabrata* ATCC2001 and *C. albicans* SC5314 (Figure 5a), while **Gua‐SMACS‐16**’s MIC_90_ values against these species were 3.9, 125, and >1000 µg/mL, respectively (Figure 2a). This MIC‐glucan correlation was also observed for a panel of *C. tropicalis* clinical isolates. As shown in Figure 5b,c, among the five clinical isolates we have in our laboratory, four showed similar or lower glucan levels than the ATCC13803 strain, and their MIC_90_ were all in the 3.9 – 7.8 µg/mL range. In contrast, the resistant strain, CW0366, displayed a glucan level higher than other *C. tropicalis* strains, but comparable to that of *C. glabrata* ATCC2001 as determined by aniline blue staining (Figure 5a,b). As expected, **Gua‐SMACS‐16**’s MIC_90_ against CW0366 was found to be 125 µg/mL, identical to that against *C. glabrata* ATCC2001. The cell wall chitin levels in these *C. tropicalis* species were also measured using both Calcofluor White (CFW) staining and the acid hydrolysis methods [52]. The results revealed comparable chitin levels across all the strains (Figure S14), suggesting that chitin plays a minimal role in governing the antifungal selectivity. We further compared the antifungal effect against *C. albicans* SC5314 and its isogenic mutant *gsc1*:Δ. *GSC1* is an essential gene that encodes a subunit of 1,3‐β‐glucan synthase. As a result, the mutant contains a much lower 1,3‐β‐glucan content in the cell wall than wild type [50]. The MIC_50_ of **Gua‐SMACS‐16** were >1000 and 125 µg/mL, respectively, against these two strains, providing additional experimental support for the role of β‐glucan in determining sensitivity to **Gua‐SMACS‐16**.

We hypothesized that the highly crosslinked glucans, including both 1,3‐ and 1,6‐β‐glucan, can serve as a barrier to block the entry of **Gua‐SMACS‐16** molecules, hence protect the underlying cytoplasmic membrane. Due to the low β‐glucan content in the *C. tropicalis* cell wall, its cytoplasmic membrane is more accessible and hence becomes sensitive to **Gua‐SMACS‐16**. In this context, exogenous glucans added to the culture should be able to compete with the cell wall for **Gua‐SMACS‐16** binding and thus alleviate the antifungal effect. As shown in Figure 5d, in the presence of 31.3 and 125 µg/mL glucan isolated from baker's yeast *S. cerevisiae*, **Gua‐SMACS‐16**’s MIC_90_ value against *C. tropicalis* ATCC13803 increased from 3.0 µg/mL to 5.0 and 8.0 µg/mL, respectively. Interestingly, in the presence of 125 µg/mL laminarin or pustulan (i.e., different types of glucans from non‐fungal sources), the MIC_90_ value only increased modestly to 4.0 µg/mL, potentially due to glucan structural differences. It is also worth noting that the addition of other polysaccharide components commonly found in the fungal cell wall, such as chitin or mannan, did not induce any MIC increase, suggesting a highly specific interaction between **Gua‐SMACS‐16** and β‐glucan in the fungal cell wall.

### 2.7 Ultra‐Strong Synergy With Caspofungin

Our finding that **Gua‐SMACS‐16** interacts with β‐glucan suggests a possible synergy with caspofungin, a relatively new antifungal drug in the echinocandin family, first approved by FDA in 2001 [53]. Caspofungin exerts its effect mainly by inhibiting the 1,3‐β‐glucan synthase and hence weakening the fungal cell wall [54]. We hypothesized that fungal cells treated with sublethal concentrations of caspofungin would have less 1,3‐β‐glucan in their cell wall, therefore exposing the underlying cytoplasmic membrane to **Gua‐SMACS‐16** attack. As shown in Figure 5e, an ultra‐strong synergy between the two antifungal agents was observed in inhibiting the growth of *C. albicans* SC5314 in a checkerboard assay. The MIC_90_ values for **Gua‐SMACS‐16** and caspofungin alone were >1000 µg/mL (Figure 2a) and 62.5 ng/mL, respectively. In stark contrast, when combined, only 2.0 µg/mL of **Gua‐SMACS‐16** and 2.0 ng/mL of caspofungin were needed to inhibit >90% of cell growth. The fractional inhibitory concentration index (FICI) was calculated to be lower than 0.034, which is below the FICI ≤ 0.05 benchmark for defining ultra‐synergy [55]. The PI dye assay and MFC experiments further confirmed that the synergistic effect is membrane‐disrupting and fungicidal (Figure S15). Such synergistic effects were similarly observed from checkerboard assays conducted in other common culture media, such as cation‐adjusted Muller Hinton Broth (MHB) and RPMI1640 (Figures S16 and S17), but abolished in the presence of serum, suggesting a need for appropriate drug delivery systems (e.g., liposomes, polymeric nanoparticles) for systemic fungal infection treatment. Similar checkboard assay was also performed in the presence of BSA up to 250 µg/mL or additional ionic strength up to 100 mM NaCl, where the synergy between **Gua‐SMACS‐16** and caspofungin against the model *C. albicans* SC5314 was maintained under both conditions (Figures S18 and S19), indicating that protein binding or ionic strength change is unlikely to account for the loss of synergy observed in serum. This finding is also consistent with our earlier observation that additional NaCl or BSA in the culture media does not significantly affect the antifungal activity of **Gua‐SMACS‐16** as a single agent against *C. tropicalis* ATCC13803 (Figures S3 and S4). Notably, strong synergy was similarly observed against other FPPL‐listed *Candida* species including the recently emerged superbug *C. auris* from different clades (Figure 5f,g; Figures S20–S22). In other words, the presence of caspofungin transforms **Gua‐SMACS‐16** from a highly *C. tropicalis*‐selective antifungal into a pan‐active formulation effective against various critical fungal pathogens.

For other newer members in the echinocandin family, anidulafungin demonstrated a comparable synergistic effect with **Gua‐SMACS‐16** (Figure S23), but not micafungin. This is likely due to strong electrostatic interactions between the cationic **Gua‐SMACS‐16** and anionic micafungin molecules, resulting in precipitation immediately after mixing (Figure S24a). In clear contrast, inter‐molecular interaction was not observed between **Gua‐SMACS‐16** and caspofungin, as experimentally confirmed by NMR spectroscopy (Figure S24b), suggesting that the antifungal synergy arises from targeting different cellular components. One hypothesis is that caspofungin reduces the β‐glucan level in the cell wall and hence allows more **Gua‐SMACS‐16** molecules to diffuse through and disrupt the underlying cytoplasmic membrane [36]. Meanwhile, the compromised membrane may impair the membrane‐bound 1,3‐β‐glucan synthase, the inhibitory target of caspofungin, rendering the affected *Candida* cells more sensitive to caspofungin [56]. In support of this hypothesis, a synergistic effect was also observed between caspofungin and Triton X‐100 (Figure S25), a common nonionic membrane‐disrupting surfactant [57].

### 2.8 Generalizability of the Glucan‐Dependent Antifungal Activity

Building upon the serendipitous discovery of **Gua‐SMACS‐16**’s glucan‐dependent antifungal potency, selectivity, and remarkable synergy with echinocandins, we sought to determine whether similar applies for other guanidinium‐containing antifungal designs that have been extensively reported in the literature [28]. In a recent study [14], we described a series of related cationic supramolecules featuring a POSS core but with only eight or ten peripheral guanidinium groups, and they displayed moderate antifungal activity against *C. albicans*. For instance, **Gua‐SMACS‐8** (previously referred to as **T8‐C3** [14], structure shown in Figure S26), exhibited a MIC_90_ of 62.5 µg/mL against *C. albicans* ATCC10231. Here, we expanded the antifungal testing panel to multiple other *Candida* species and found a consistent selectivity toward *C. tropicalis*. The MIC_90_ against *C. tropicalis* ATCC 13803 was 2.0 µg/mL, whereas the values for others were 62.5 µg/mL or higher. Interestingly, **Gua‐SMACS‐8** also exhibited a similar strong synergistic antifungal effect with caspofungin across various FPPL‐listed *Candida* species (Figures S27–S31), supporting the generality of the glucan‐dependent antifungal effect. As noted earlier, guanidinium functionalization is preferable over other simpler cationic amine moieties for antimicrobial use. To show the essential role of guanidinium for antifungal activity in our SMACS design, we measured the MIC_90_ of a control compound containing a POSS core and eight primary ammonium groups (**PA‐SMACS‐8**, structure shown in Figure S32), and the values were all greater than or equal to 125 µg/mL.

## 3 Conclusions

In summary, we present here a novel class of giant guanidinium‐perfunctionalized supramolecular spheres with potent antifungal activity against and high selectivity to *Candida tropicalis* (MIC = 3.9‐7.8 µg/mL, 0.4‐0.8 µM), while sparing other closely related *Candida* species, non‐*Candida* fungal, bacterial, or mammalian cells. The antifungal effect is mainly attributed to the disruption of cytoplasmic and organelle membranes, and the unprecedented high selectivity to *C. tropicalis* is ascribed to its low cell wall β‐glucan content, which fails to act as a barrier for the membrane‐disrupting supramolecules. Notably, the design exhibits ultra‐strong synergistic effects with caspofungin, a clinical antifungal targeting 1,3‐β‐glucan synthase, against all tested *Candida* species including the intractable *C. auris*, lowering the required effective doses by orders of magnitude. Collectively, our serendipity discovery underscores the critical role of cell wall glucan in mediating the antifungal effects of AFP mimetics, establishes a new design strategy for species‐specific antifungal agents, and meanwhile unveils a novel approach to developing potent synergizers for next‐generation antifungal combination therapy.

## Conflicts of Interest

The authors declare no conflicts of interest.

## Supporting information



The supplementary materials include chemical synthesis and characterization details, experimental procedures, and supplementary data. The authors have cited additional references within the Supporting Information [14, 58, 59, 60, 61, 62, 63, 64, 65, 66].**Supporting File**: advs75480‐sup‐0001‐SuppMat.docx.

## Data Availability

The data that support the findings of this study are available from the corresponding author upon reasonable request.
