# Supplementary material for: A Cationic Supramolecule With Potent Antifungal Activity, Single‐Species Selectivity, and Strong Synergy With Echinocandins
Source: Adv Sci (Weinh). 2026 Apr 29;13(41):e75480. doi: 10.1002/advs.75480 (PMC13335614; doi:10.1002/advs.75480)
Supplement: Supplementary file 1 — The supplementary materials include chemical synthesis and characterization details, experimental procedures, and supplementary data. The authors have cited additional references within the Supporting Information [14, 58, 59, 60, 61, 62, 63, 64, 65, 66].Supporting File: advs75480‐sup‐0001‐SuppMat.docx. [file ADVS-13-e75480-s001.docx]

**Supporting Information**

**A Cationic Supramolecule with Potent Antifungal Activity, Single-Species Selectivity, and Strong Synergy with Echinocandins**

Tianjiao Dai, Adrielle Xianwen Chen, Eve Wai Ling Chow, Li Mei Pang, Jianguo Li, Chandra Shekhar Verma, Stefan H. Oehlers, Yue Wang,* and Ning Li*

Corresponding authors: [ywangcandida@gmail.com](mailto:ywangcandida@gmail.com) (YW); [li_ning@a-star.edu.sg](mailto:li_ning@a-star.edu.sg) (NL)

**General Remarks**

All chemicals and reagents were purchased from commercial suppliers and used as received unless otherwise stated. Microbial strains were obtained from commercial sources or clinical collaborators. Flash column chromatography was performed using pre-coated 0.2-mm silica. Chemical yield refers to pure isolated substances. ^1^H and ^13^C NMR spectra were recorded on a Bruker ACF-400 (400 MHz) spectrometer. The solvent signals of CDCl_3_ and DMSO-*d_6_* were referenced at δ = 7.26 and 2.50 ppm, respectively. ^1^H NMR data were recorded in the order: chemical shift value, multiplicity (s, singlet; d, doublet; t, triplet; q, quartet; m, multiplet; br, broad; etc.), and number of protons that gave rise to the signal, where applicable. ^13^C NMR spectra are proton-decoupled and recorded on Bruker ACF-400 (101 MHz). The solvents CDCl_3_ and DMSO-*d_6_* were referenced at δ = 77.2 and 39.5 ppm, respectively. CDCl_3_ (99.8%-deuterated) and DMSO-*d_6_* (99.5%-deuterated) were purchased from Cambridge Isotope Laboratories, Inc. and used as received without further purification. GPC was recorded on a Waters 2695D separation module equipped with an Optilab rEX differential refractometer (Wyatt Technology Corporation) and a Waters HR-4E column. HPLC was carried out using Agilent 1260 Infinity HPLC system on an Atlantis HILIC Silica column. The MALDI-TOF analysis was performed using a JMS-S3000 MALDI-TOF MS system.

**Synthetic Steps and Chemical Structures**


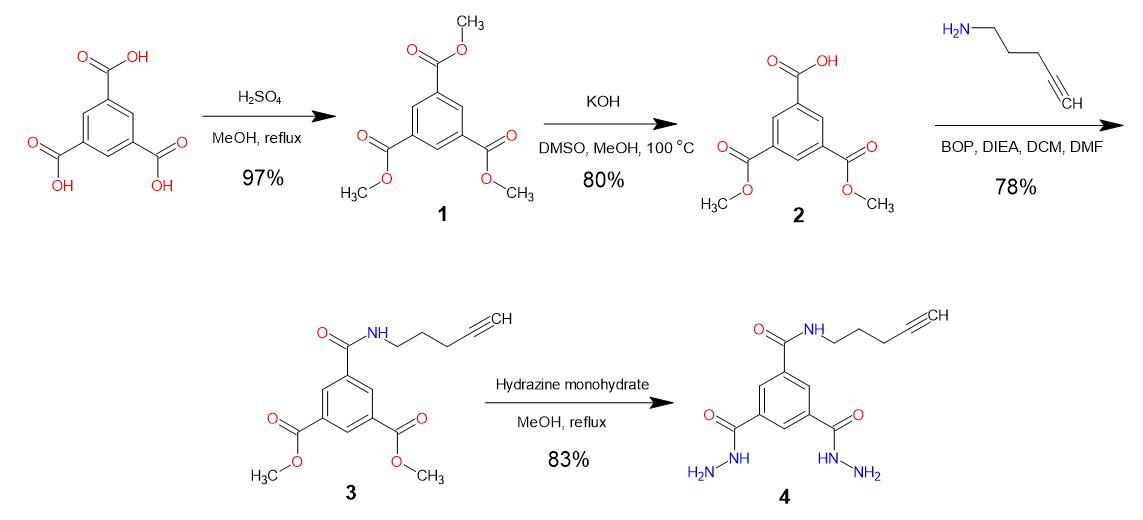


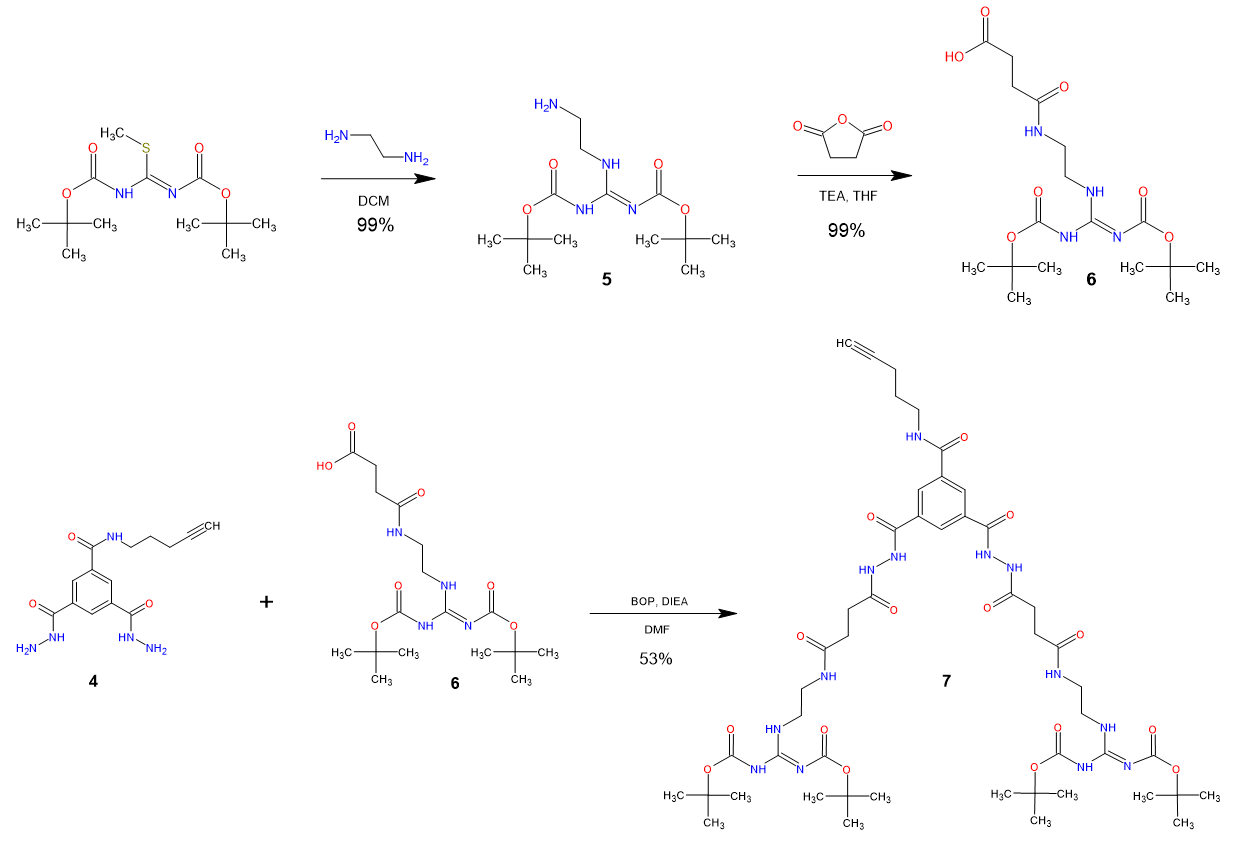


**Scheme S1.** Synthetic steps for the Y-shaped arms containing two Boc-protected guanidine groups and one alkyne moiety.


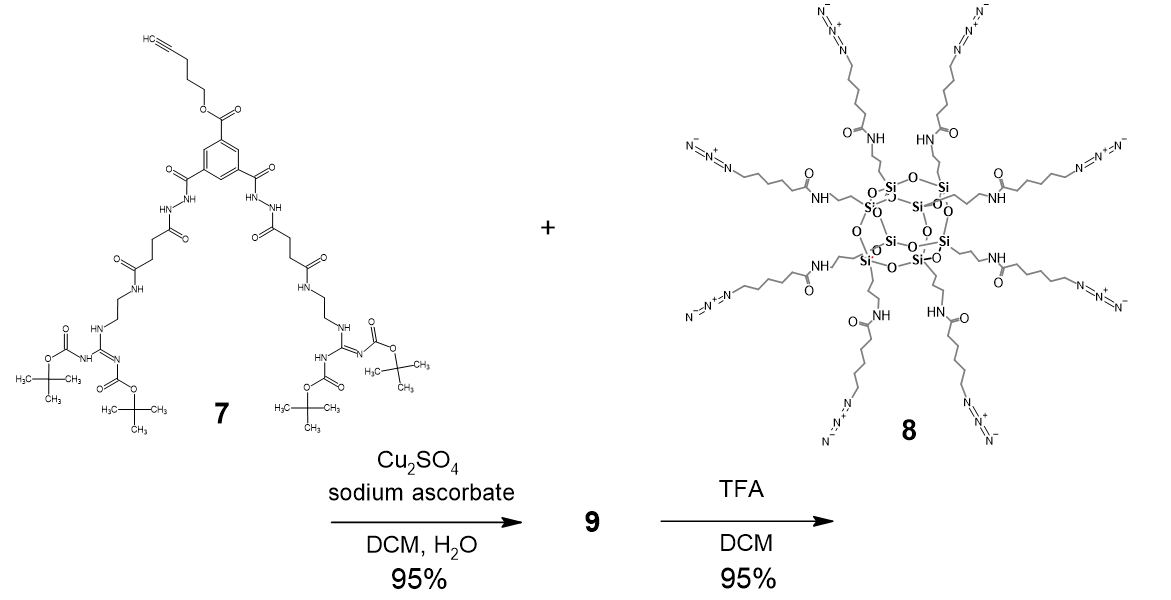


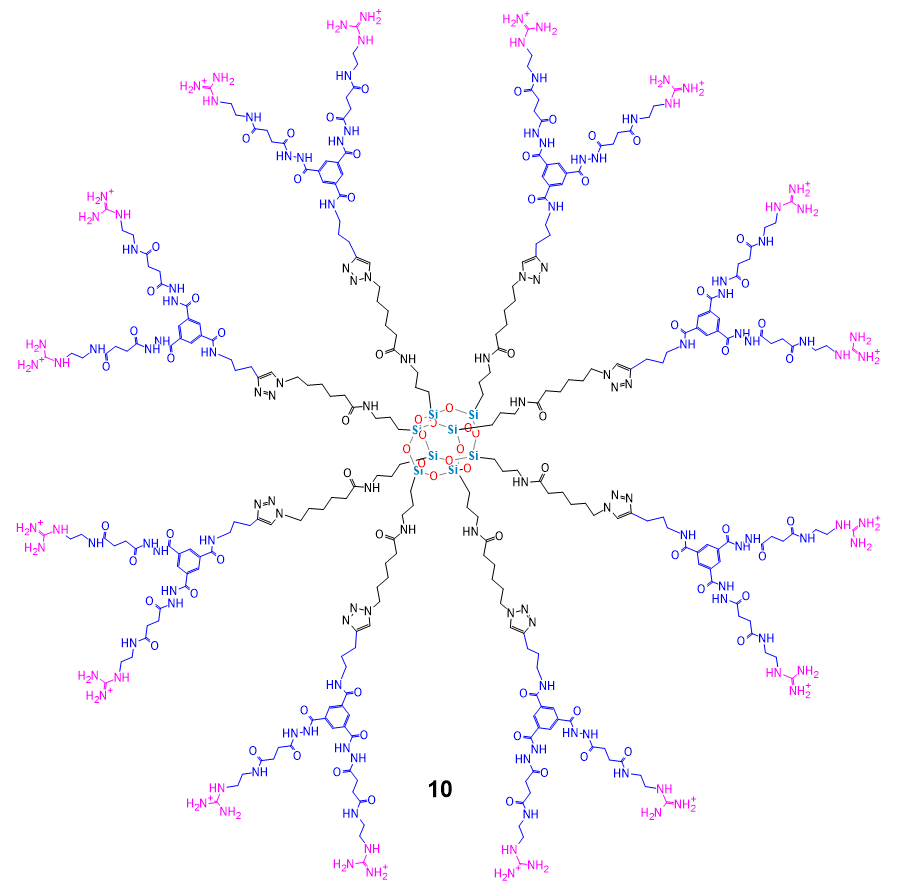


**Scheme S2.** Click chemistry to produce the molecular sphere and subsequent TFA deprotection to afford **Gua-SMACS-16** (compound **10**).


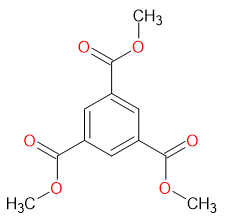
**Synthetic Procedures and Compound Characterization**

Compound **1**: Trimesic acid (2.1 g, 10 mmol) was suspended in 120 mL methanol, followed by careful addition of 15 mL concentrated sulfuric acid. The reaction mixture was heated under reflux overnight at 85^o^C. After cooling to room temperature, methanol was removed *in vacuo* before adding in dichloromethane to redissolve the crude product. The crude product was washed with deionized water three times and dried over sodium sulfate. Removal of dichloromethane *in vacuo* gave desired product **1** as white solid. Yield: 2.46g, 97%. ^1^H NMR (400 MHz, CDCl_3_) δ 8.85 (s, 3H), 3.97 (s, 9H). ^13^C NMR (101 MHz, CDCl_3_) δ 165.6, 134.8, 131.4, 52.8.


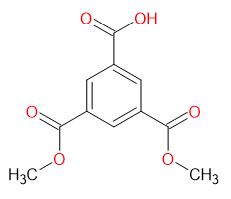
Compound **2**: Compound **1** (504 mg, 2 mmol) was dissolved into 10 mL dimethyl sulfoxide and heated to 100 ^o^C. Potassium hydroxide (KOH, 112.2 mg, 2 mmol) was dissolved into 1.5 mL methanol and the solution was added to the reaction mixture while stirring. After 1 hour, the reaction mixture was cooled to room temperature before adding 30 mL deionized water. Extraction using diethyl ether was performed to remove unreacted precursors, followed by acidification of the aqueous phase using sulfuric acid (4% in water). The acidified aqueous phase was further extracted using dichloromethane. The combined organic phase was washed four times using deionized water and dried over sodium sulfate. Removal of solvent *in vacuo* produced desired product **2** as white solid. Yield: 380 mg, 80%. ^1^H NMR (400 MHz, DMSO-*d_6_*) δ 13.7 (br, 1H), 8.66 (d, 2H), 8.63 (t, 1H), 3,93 (s, 6H). ^13^C NMR (101 MHz, DMSO-*d_6_*) δ 162.6, 164.7, 133.7, 133.1, 132.3, 130.9, 52.8.


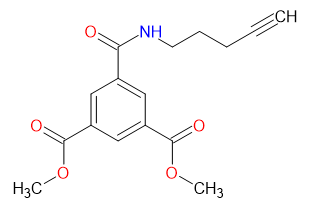
Compound **3**: Compound **2** (238 mg, 1 mmol), 4-pentyn-1-amine hydrochloride (120 mg, 1 mmol), benzotriazol-1-yloxytris(dimethylamino)phosphonium hexafluorophosphate (BOP, 486 mg, 1.1 mmol) were dissolved in a mixture solvent of dichloromethane (8 mL) and dimethylformamide (2 mL). N,N-Diisopropylethylamine (426 mg, 3.3 mmol) was further added into the clear reaction mixture. After overnight reaction at room temperature, solvent was removed *in vacuo* and residue was re-dissolved in dichloromethane and washed with deionized water three times. The crude product was subject to column purification using 2 – 5% methanol in dichloromethane. Pure product of compound **3** was obtained as pale-yellow solid. Yield: 237 mg, 78%. ^1^H NMR (400 MHz, DMSO-*d_6_*) δ 8.95 (t, 1H), 8.67 (d, 1H), 8.57 (t, 1H), 3.93 (s, 6H), 3.37 (q, 2H), 2.8 (t, 1H), 2.24 (td, 1H), 1.81 – 1.67 (m, 1H). ^13^C NMR (101 MHz, DMSO-*d_6_*) δ 165.0, 164.2, 135.6, 132.1, 131.7, 130.5, 84.1, 71.4, 52.7, 38.7, 27.9, 15.5.


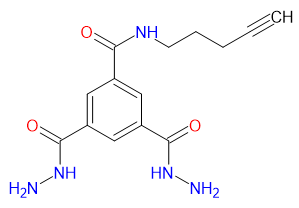
Compound **4**: Compound **3** (303 mg, 1 mmol) was dissolved in 12 mL methanol to form clear a solution before 3 mL hydrazine hydrate was added dropwise. The reaction mixture was heated under reflux overnight at 85^o^C. After cooling the reaction to room temperature, solvent was removed *in vacuo* and the crude product was washed with cold ethanol to afford pure compound **4** as white solid. Yield: 253 mg, 83%.^1^H NMR (400 MHz, DMSO-*d_6_*) δ 9.86 (s, 2H), 8.65 (t, 1H), 8.35 (d, 2H), 8.32 (t, 1H), 4.57 (s, 4H), 2.81 (t, 1H), 2.24 (td, 2H), 1.79 – 1.67 (m, 2H). ^13^C NMR (101 MHz, DMSO-*d_6_*) δ 165.4, 165.1,
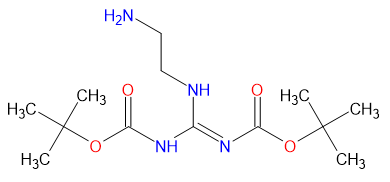
135.0, 133.8, 128.3, 128.1, 84.1, 71.4, 38.5, 28.0, 15.5.

Compound **5**: 1,3-bis(tert-butoxycarbonyl)-2-methyl-2-thiopseudourea (1.45 g, 5 mmol) was dissolved in 7.5 mL dichloromethane. The solution was added dropwise into another clear solution containing ethylenediamine (751 mg, 12.5 mmol) dissolved in 10.7 mL dichloromethane. After 4 hours at room temperature, the reaction mixture was washed with brine three times and dried over sodium sulfate. Removal of solvent *in vacuo* gave desired compound **5** in pure form. Yield: 1.5 g, 99%. ^1^H NMR (400 MHz, CDCl_3_) δ 11.5 (s, 1H), 8.64 (s, 1H), 3.47 (q, 2H), 2.88 (t, 2H), 1.51 – 1.48 (b, 18H). ^13^C NMR (101 MHz, CDCl_3_) δ 163.8, 156.6, 153.4, 83.3, 79.4, 43.6, 41.1, 28.5, 28.3.


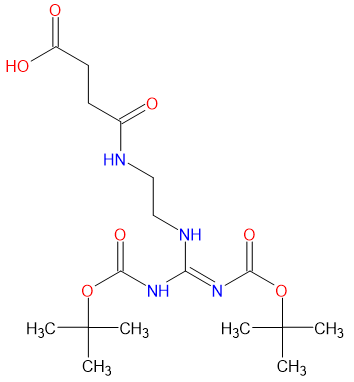
Compound **6**: Compound **5** (1.48 g, 4.9 mmol), succinic anhydride (589 mg, 5.9 mmol), and triethylamine (746 mg, 7.4 mmol) were dissolved in 30 mL tetrahydrofuran. After an overnight reaction, 30 mL of deionized water was added to the reaction mixture, and the reaction was stirred for another hour. Removal of tetrahydrofuran produced a white cloudy suspension, to which 20 mL deionized water and 20 mL potassium bisulfate solution (5% in deionized water) were added. Product was extracted using dichloromethane and dried over sodium sulfate. Removal of the solvent *in vacuo* gave the pure product of **6** as white solid. Yield: 1.96g, 99%. ^1^H NMR (400 MHz, DMSO-*d_6_*) δ 12.05 (br, 1H), 11.48 (s, 1H), 8.36 (t, 1H), 8.00 (t, 1H), 3.37 (b, 2H), 3.22 – 3.14 (m, 2H), 2.41 (t, 2H), 2.30 (t, 2H), 1.47 (s, 9H), 1.39 (s, 9H). ^13^C NMR (101 MHz, DMSO-*d_6_*) δ 173.8, 171.3, 163.1, 155.7, 151.9, 82.8, 78.2, 40.2, 38.1, 30.1, 29.2, 28.0, 27.6.


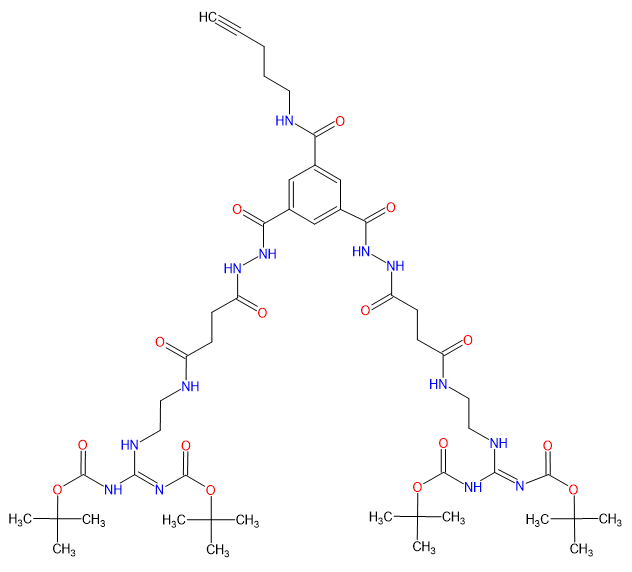
Compound **7**: Compound **4** (212 mg, 0.7 mmol), compound **6** (563 mg, 1.4 mmol), benzotriazol-1-yloxytris(dimethylamino)phosphonium hexafluorophosphate (BOP, 752 mg, 1.7 mmol), and N,N-Diisopropylethylamine (597 mg, 4.6 mmol) were dissolved in 15 mL anhydrous dimethylformamide. After three days of reaction at room temperature, solvent was removed *in vacuo* and residue was re-dissolved in dichloromethane and washed with deionized water three times. The crude product was subject to column purification using 5% methanol in dichloromethane. Pure product of compound **7** was obtained as pale-yellow solid. Yield: 400 mg, 53%. ^1^H NMR (400 MHz, DMSO-*d_6_*) δ 11.48 (s, 2H), 10.52 (s, 2H), 10.03 (s, 2H), 8.74 (t, 1H), 8.50 – 8.41 (m, 3H), 8.38 (t, 2H), 8.05 (t, 2H), 3.41 – 3.33 (m, 4H), 3.24 – 3.16 (m, 4H), 2.80 (t, 1H), 2.49 – 2.42 (m, 4H), 2.41 – 2.34 (m, 4H), 2.24 (td, 2H), 1.80 – 1.65 (m, 2H), 1.49 – 1.36 (m, 40H). ^13^C NMR (101 MHz, DMSO-*d_6_*) δ 171.7, 171.2, 165.7, 165.1, 163.5, 156.1, 152.4, 135.7, 133.6, 129.8, 129.5, 83.6, 83.3, 78.7, 71.9, 39.1, 38.5, 30.9, 29.2, 28.5, 28.1, 16.0.

Compound **9**: Compound **7** (292 mg, 0.27 mmol), compound **8** (68.2 mg, 0.034 mmol, reported previously in *ACS Appl. Mater. Interfaces*, **2023**, *15*, 354-363), CuSO_4_·5H_2_O (1.7 mg, 6.8 µmol), and sodium ascorbate (4 mg, 20 µmol) were suspended in a biphasic solvent system of water (15 mL) and dichloromethane (15 mL). The reaction mixture was stirred at room temperature for three days with protection from light. The reaction mixture was washed with water three times, then dried over sodium sulfate and evaporated *in vacuo* to afford the pure product of **9** as a pale-yellow solid. Yield: 341 mg, 95%. ^1^H NMR (400 MHz, DMSO-*d_6_*) δ 11.48 (s, 16H), 10.52 (s, 16H), 10.03 (s, 16H), 8.78 (t, 8H), 8.50 – 8.42 (m, 24H), 8.37 (t, 16H), 8.04 (t, 16H), 7.85 (s, 8H), 7.78 (s, 8H), 4.25 (t, 16H), 3.19 (m, 32H), 2.99 (br, 16H), 2.66 (t, 16H), 2.50 – 2.41 (m, 32H), 2.41 – 2.32 (m, 32H), 2.10 – 2.00 (m, 16H), 1.94 – 1.81 (m, 16H), 1.80 – 1.69 (m, 16H), 1.55 – 1.33 (m, 352H), 1.26 – 1.15 (m, 16H), 0.57 (br, 16H).


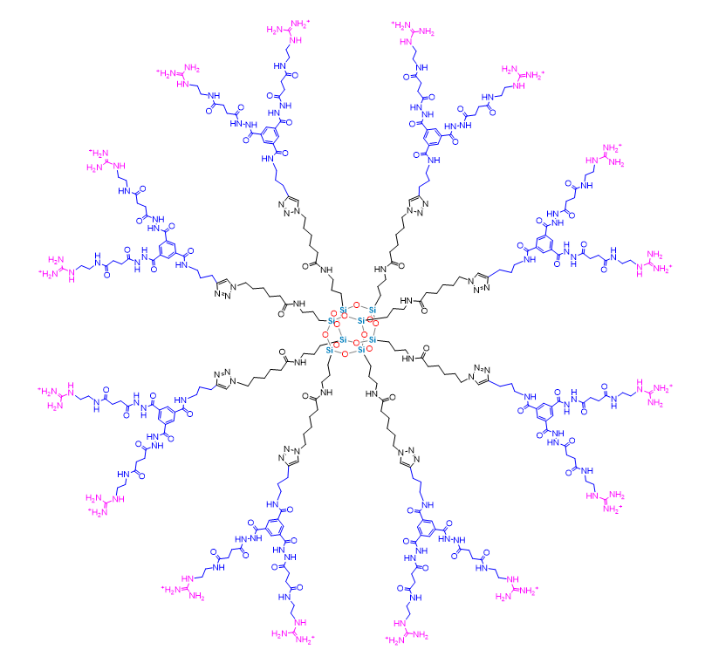
Compound **10**: Compound **9** (370 mg, 35 µmol) and trifluoroacetic acid (5 mL) was dissolved in 30 mL anhydrous dichloromethane. After overnight reaction at room temperature under nitrogen protection, solvent was removed *in vacuo*. The crude product was dissolved in methanol and precipitated using diethyl ether to afford desired product **10** as pale-yellow solid. Yield: 358 mg, 95%. ^1^H NMR (400 MHz, DMSO-*d_6_*) δ 10.54 (s, 16H), 10.06 (s, 16H), 8.80 (s, 8H), 8.54 – 8.39 (m, 24H), 8.08 (s, 16H), 7.92 – 7.77 (m, 16H), 7.64 – 6.95 (m, 80H), 4.26 (t, 16H), 3.17 (br, 80H), 2.99 (br, 16H), 2.67 (t, 16H), 2.50 – 2.43 (m, 32H), 2.43 – 2.36 (m, 32H), 2.04 (t, 16H), 1.87 (t, 16H), 1.77 (t, 16H), 1.57 – 1.37 (m, 32H), 1.26 – 1.16 (m, 16H), 0.58 (br, 16H). ^29^Si NMR (500 MHz, DMSO-*d_6_*) δ -65.75. LC-MS: [M – 16 TFA + 2 Na^+^ + 4 H^+^]^22+^ cald. 337.84, found 337.85; [M – 16 TFA + 3 Na^+^ + 2 H^+^]^21+^ cald. 354.93, found 355.25.

**MIC and MFC Measurements**

The minimum inhibitory concentration (MIC) was measured using the broth microdilution method adapted from the CLSI protocol. In brief, *Candida* cells were first cultured overnight at 30 ^o^C overnight with 220 rpm shaking in YPD. The cells were washed two times using sterile PBS before adjusting optical density (OD) reading to ∼0.08 at 660 nm in the same, which corresponds to a concentration of ∼1×10^6^ CFU/mL. The suspension was then further diluted 1000-fold using synthetic complete (SC) media to achieve an inoculum of ∼1×10^3^ CFU/mL. SC media (100 μL) containing **Gua-SMACS-16** (with a fixed DMSO concentration of 0.5%, v/v) at desired concentrations was loaded into each well of a 96-well microplate. An equal volume of fungal suspension (∼1×10^3^ CFU/mL) in SC media was added into each well. The plates were incubated at 30 ^o^C for 48 h. The MIC was defined as the lowest concentration at which there was no fungal growth observed with naked eyes and the microplate reader. SC media containing fungal cells alone was used as the negative control, and each test was conducted with at least three parallel replicates. The suspension with no visible fungal growth was spread on YPD agar plates for quantification, and MFC was determined as the lowest concentration wherein no colonies from surviving cells were observed.

**Haemolysis Assay**

The haemolytic activity of **Gua-SMACS-16** was tested using fresh red blood cells of C57BL/6 mice. In brief, fresh blood was diluted 30-fold in sterile PBS to achieve 3.3% v/v of blood content. The compound was dissolved in sterile PBS with a concentration range of 0−2000 μg/mL. Equal volume of **Gua-SMACS-16** solution was then mixed with the diluted blood sample (100 μL each). The mixtures were then incubated at 37 °C for 1 h to allow for a thorough interaction between the red blood cells and **Gua-SMACS-16** molecules. Afterward, the mixture was subject to centrifugation (1000*g* for 5 min, 4 °C), and 100 μL aliquots of the supernatant were transferred into a new 96-well microplate. The haemoglobin release was measured spectrophotometrically by recording the absorbance at 576 nm using the microplate reader (Tecan, Switzerland). Two control groups were employed for this assay: untreated red blood cell suspension as the negative control and blood cell suspension treated with 0.8% Triton-X as the positive control. The percentage of haemolysis was defined as Haemolysis (%) = [(OD_576_ of the treated sample − OD_576_ of the negative control)/(OD_576_ of the positive control − OD_576_ of the negative control)] × 100%. Each assay was performed in six parallel replicates, and the average values were reported with standard deviations shown as error bars.

**Cell Viability Test**

*In vitro* cytotoxicity of **Gua-SMACS-16** was tested against L929 mouse fibroblast cells. In brief, ∼5 × 10^3^ cells were first seeded into wells of a black 96-well plate and incubated for 24 h at 37 °C. **Gua-SMACS-16** was dissolved in media with a 2-fold serial dilution to reach the desired concentrations. The spent media above the seeded cell monolayer was removed carefully, followed by adding 100 μL of the **Gua-SMACS-16** solution to each well. After incubation for another 48 h at 37 °C, the solution was removed, and 100 μL alamar blue and DMEM mixture (1:9, v/v) was added in each well. After 3 h incubation at 37 °C in the dark, the fluorescence intensity of alamar blue was recorded using a microplate reader (Tecan, Switzerland) with 560 nm excitation and 590 nm emission wavelengths. Two controls were used in this assay: cells with alamar blue and alamar blue only. The percentage of cell viability was defined as Viability (%) = [(F_590_ of the treated well − F_590_ of alamar blue only)/(F_590_ of untreated cells with alamar blue − F_590_ of alamar blue only)] × 100%. Each assay was performed in eight parallel replicates, and the average values were reported with standard deviations shown as error bars.

**Biofilm Disruption Assay**

*C. tropicalis* ATCC13803 cells were cultured in SC media at 30 °C with 220 rpm shaking overnight. The cells were harvested and washed twice using sterile PBS. About 2×10^5^ cells (in 120 μL SC media) were loaded into each well of a transparent flat-bottom 96-well micro plate followed by incubation at 37 °C with 75 rpm shaking for 24 h. The spent media was removed, and another 120 μL fresh SC media was added for a new cycle of biofilm development. Afterwards, the biofilms were thoroughly washed with sterile PBS, and **Gua-SMACS-16** solution at the desired concentration in SC media was then added to each well, followed by incubation for another 24 h at 30 °C with 75 rpm shaking for biofilm disruption. At the end of the treatment, spent media was removed carefully, and the biofilm residue was washed with 200 μL of sterile PBS thrice to remove planktonic cells. Crystal violet (CV) staining assay was used to quantify the biofilm biomass. In brief, 200 μL of methanol was added to each well to fix the biomass for 15 min, followed by adding 200 μL of crystal violet solution (1%, w/v) and incubation for another 10 min for staining. The excess crystal violet was washed away with DI water. The crystal violet bound with the biomass residue was extracted by 33% glacial acetic acid (200 μL) and absorbance at 570 nm was recorded using the microplate reader (Tecan, Switzerland). Each test was performed in eight parallel replicates, and the average values were reported with standard deviations shown as the error bars.

**Acute Toxicity to Zebrafish Embryos**

Zebrafish embryos were produced by natural spawning under A*STAR IACUC Approval 211667 and raised at 28 °C in E3 embryo media supplemented with methylene blue. Embryos were dechorionated at one day post fertilization and distributed to treatment groups at two days post fertilization at a density of approximately 20 embryos in 5 ml of media per well in 6 well plates. Compounds were added directly into the E3 embryo media and survival was monitored visually by heartbeat and response to touch assays.

**PI Assay to Monitor Membrane Permeability**

The propidium iodide (PI) dye assay was performed to assess fungal cell membrane permeability upon treatment.^[1]^ In brief, *C. tropicalis* ATCC13803 cells were cultured at 30 °C with 220 rpm shaking for 24 h in SC media, before being harvested, washed, and resuspended in a buffer solution containing 5 mM glucose and 5 mM HEPES at pH 7.2 to an OD_600_ value of ∼0.3. This microbial suspension (150 μL) was added to wells of a black 96-well plate. PI solution (50 μM, 10 μL, in sterile water) was added and preincubated for 10 min. Following preincubation, fluorescence was measured for the next 8 min with a time interval of 2 min using a microplate reader (535 nm excitation, 617 nm emission, Tecan, Switzerland). **Gua-SMACS-16** at desired concentrations was then added, and the fluorescence intensity was monitored with a time interval of 2 min. Each test was performed in four parallel replicates, and the average values were reported with standard deviations shown as the error bars.

**PI and FM4-64 Staining and Fluorescence Imaging**

*C. tropicalis* ATCC13803 was pre-cultured in YPD overnight at 30°C with shaking at 220 rpm. The following day, cells were washed twice with PBS, adjusted to the density of ~1.5x10^7^ cells/mL in the same, and then treated with **Gua-SMACS-16** at a concentration of 62.5 µg/mL for 4 hours at room temperature. Cells incubated in PBS without treatment served as untreated control. After incubation, the fungal cells were washed with sterile PBS, and then stained with either Propidium Iodide (PI, in PBS, 10 µM, 20 min, room temperature) or FM4-64 (4 µM, in YPD, 1 h, 30°C), respectively. Cells stained with PI were washed with PBS. Cells stained with FM4-64 were washed and incubated in fresh YPD (1 h, 30°C) and then washed again with PBS. The stained cells were concentrated and 3 µL aliquots were mounted onto microscope slides and covered with a coverslip. Fluorescence imaging was performed using an upright fluorescence microscope (Ni-E, Nikon). Cells stained with PI were imaged under bright field and Cy3 channel (λ_ex_ = 550 nm, λ_em_ = 645 nm). Cells stained with FM4-64 were imaged under bright field and Cy5 channel (λ_ex_ = 621 nm, λ_em_ = 700 nm).

**Aniline Blue Staining and β-1,3-Glucan Quantification**

All the tested strains were pre-cultured in YPD overnight at 30°C with shaking at 220 rpm. On the next day, the cells were washed twice with sterile PBS and adjusted to OD_660_ = 1. The cells were then incubated with 1 mg/mL aniline blue within a 96-well plate, and the fluorescence intensity (λ_ex_ = 375 nm, λ_em_ = 492 nm) was measured with a microplate reader (Infinite 200, Tecan).

**Molecular Dynamics Simulation**

Molecular dynamics simulations were conducted to understand the mode of interactions of **Gua-SMACS-16** with a model fungal plasma membrane patch consisting of 288 lipids with a POPC:POPE:POPS:ergosterol (ERG) ratio of 4:2:1:2. The coordinates of the fungal membrane were taken from our previous study.^[2]^ One **Gua-SMACS-16** molecule with random orientation were initially placed close to the membrane and then solvated with water molecules. 0.15 M counter ions were added to neutralize the system. To remove bad atomic contacts, 500 steps of energy minimization using steep descent algorithm was performed, followed by 100 ps NPT simulations to relax the system. Three replicates were run for 1000 ns each using the Amber14sb force field. The Amber14sb force field was used to model both **Gua-SMACS-16** and the membrane, and water molecules were modelled using the TIP3P model. The atomic parameters for the silica core of **Gua-SMACS-16** were taken from a previous study, ^[3,4]^ while the parameters for the molecules including ergosterol were obtained using Antechamber and tLEaP modules of the Amber20 package.^[5]^ In each simulation, the LJ potential and the short-range electrostatic interactions were calculated using a cut-off value of 1.0 nm, while the long-range electrostatic interactions were calculated using PME. ^[6]^ Covalent bonds involving hydrogen atoms were constrained using the LINCS algorithm, enabling a time step of 2 fs to be used. The simulations were run in the NPT ensemble, with temperature maintained at 310 K using the V-rescale method ^[7]^ and the pressure maintained at 1 bar using the Parrinello-Rahman method ^[8]^ with semi-isotropic pressure coupling. All simulations were carried out using the GROMACS package.^[9]^

**Supplementary Figures**

**Figure S1**. Radius of gyration calculated using MD simulation for **Gua-SMACS-16**, and its lipophilic/hydrophilic components: (a) in water and (b) on the fungal membrane model.

**Figure S2**. MIC_90_ of **Gua-SMACS-16** against other common opportunistic non-*Candida* fungal and bacterial species present in the human microbiome.

**Figure S3**. MIC_90_ change of **Gua-SMACS-16** against *C. tropicalis* ATCC13803 in the presence of additional sodium chloride (NaCl) in the SC culture media.

**Figure S4**. MIC_90_ change of **Gua-SMACS-16** against *C. tropicalis* ATCC13803 in the presence of (a) Fetal Bovine Serum or FBS, and (b) Bovine Serum Albumin or BSA at various concentrations in SC media.

**Figure S5**. The crystal violet (CV) staining assay results showing the dose-dependent effect of **Gua-SMACS-16** against mature biofilms of *C. tropicalis* ATCC13803. * p < 0.05, ** p < 0.01, *** p < 0.001.

**Figure S6**. Acute toxicity of **Gua-SMACS-16** and comparator antifungal drugs including fluconazole (FCZ), Amphotericin B (AmB), and caspofungin (CFG) against zebrafish embryos after (a) 7 and (b) 24 hours incubation (n = 20).

**Figure S7**. DiSC_3_(5) assay results to show depolarization of cytoplasmic membrane of *C. tropicalis* ATCC13803 cells in the presence of **Gua-SMACS-16** at different concentrations.

**Figure S8**. Proximal radial distribution function (pRDF) showing that the POSS core of **Gua-SMACS-16** has a higher degree of hydration on the model membrane as compared to that in water.

**Figure S9**. Experimental MIC_90_-fold changes in **Gua-SMACS-16** against *C. tropicalis* ATCC13803 in the presence of various membrane lipids or steroids at different concentrations. The asterisk symbol indicates value greater than or equal to 125.

**Figure S10**. MIC_90_ change of **Gua-SMACS-16** against *C. tropicalis* ATCC13803 in the presence of sodium ascorbate (SA) as a reducing agent.

**Figure S11**. Experimental results using a commercial bioluminescent assay kit to detect possible generation of H_2_O_2_. The *C. tropicalis* cells used were from the ATCC13803 strain. Menadione is used as the positive control.

**Figure S12**. Fluorescence intensity of DCFH-DA after incubating 15.6 µg/mL **Gua-SMACS-16** with cells of (a) *C. tropicalis* ATCC13803, (b) *C. tropicalis* CW0271, (c) *C. tropicalis* CW0366, and (d) *C. albicans* SC5314. *** denotes p < 0.001.

**Figure S13**. Quantification of the relative cell wall porosity for different *Candida* cells cultured in YPD.

**Figure S14**. Quantification of chitin content in the cell wall of different *C. tropicalis* strains using (a) Calcofluor White (CFW) staining, and (b) acid hydrolysis method.

**Figure S15**. Time-dependent profile of PI fluorescence intensity to monitor the cell membrane permeability upon the combination treatment using **Gua-SMACS-16** and caspofungin (CFG).

**Figure S16**. Checkerboard assay results showing synergy between **Gua-SMACS-16** and caspofungin (CFG) in cation-adjusted Muller Hinton Broth against *C. albicans* SC5314. Incubation was done at 30 °C for 48 hours.

**Figure S17**. Checkerboard assay results showing synergy between **Gua-SMACS-16** and caspofungin (CFG) in RPMI1640 media supplement with 2% glucose against *C. albicans* SC5314. Incubation was done at 30 °C for 48 hours.

**Figure S18**. Checkerboard assay results showing synergy between **Gua-SMACS-16** and caspofungin (CFG) against *C. albicans* SC5314 in SC media containing (a) 125 µg/mL and (b) 250 µg/mL BSA. Incubation was done at 30 °C for 48 hours.

**Figure S19**. Checkerboard assay results showing synergy between **Gua-SMACS-16** and caspofungin (CFG) against *C. albicans* SC5314 in SC media containing additional (a) 50 mM and (b) 100 mM NaCl. Incubation was done at 30 °C for 48 hours.

**Figure S20**. Checkerboard assay results showing synergy between **Gua-SMACS-16** and caspofungin (CFG) in SC media against *C. tropicalis* CW0366. Incubation was done at 30 °C for 48 hours.

**Figure S21**. Checkerboard assay results showing synergy between **Gua-SMACS-16** and caspofungin (CFG) in SC media against *C. auris* CBS12766. Incubation was done at 30 °C for 72 hours.

**Figure S22**. Checkerboard assay results showing synergy between **Gua-SMACS-16** and caspofungin (CFG) in SC media against *C. auris* CBS12767. Incubation was done at 30 °C for 72 hours.

**Figure S23**. Checkerboard assay results showing synergy between **Gua-SMACS-16** and anidulafungin (AFG) in SC media against *C. albicans* SC5314. Incubation was done at 30 °C for 48 hours.

**Figure S24**. (a) Mixing **Gua-SMACS-16** and micafungin in water produces white precipitate immediately, likely due to the electrostatic interaction between the cationic **Gua-SMACS-16** and anionic micafungin. In comparison, no precipitate was observed for the mixture with caspofungin. (b) ^1^H NMR spectrum of **Gua-SMACS-16** and caspofungin mixture is simply a superimposition of the individual spectrum, suggesting absence of intermolecular interaction.

**Figure S25**. Checkerboard assay results showing synergy between Triton X-100 and caspofungin (CFG) in SC media against *C. albicans* SC5314.

**Figure S26**. Molecular structure of **Gua-SMACS-8** (named as **T8-C3** in a previous report,^[10]^ n = 3) and its antifungal MIC_90_ against different *Candida* species.

**Figure S27**. Checkerboard assay results showing synergy between **Gua-SMACS-8** and caspofungin (CFG) in SC media against *C. albicans* SC5314.

**Figure S28**. Checkerboard assay results showing synergy between **Gua-SMACS-8** and caspofungin (CFG) in SC media against *C. glabrata* ATCC2001.

**Figure S29**. Checkerboard assay results showing synergy between **Gua-SMACS-8** and caspofungin (CFG) in SC media against *C. auris* CBS10913.

**Figure S30**. Checkerboard assay results showing synergy between **Gua-SMACS-8** and caspofungin (CFG) in SC media against *C. auris* CBS12766.

**Figure S31**. Checkerboard assay results showing synergy between **Gua-SMACS-8** and caspofungin (CFG) in SC media against *C. auris* CBS12767.


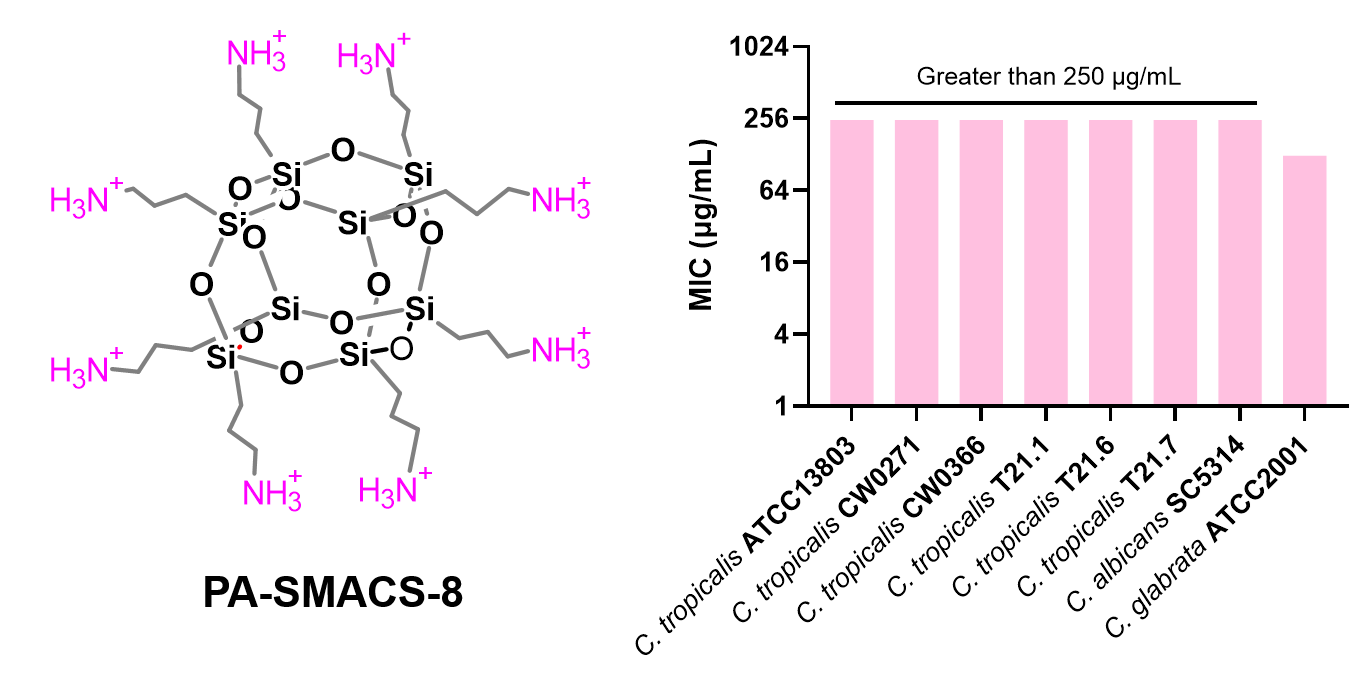


**Figure S32.** (a) Molecular structure and (b) high antifungal MIC values of **PA-SMACS-8**, showing the essential role of guanidinium functionalization.

**Supplementary Table**

**Table S1.** The MIC_90_ values of **Gua-SMACS-16** (in unit of µg/mL) against the clinical isolates of *Candida* spp. obtained from Singapore National University Hospital. Experiment was conducted in the SC media at 30 ^o^C with 48 – 72 hours of incubation.


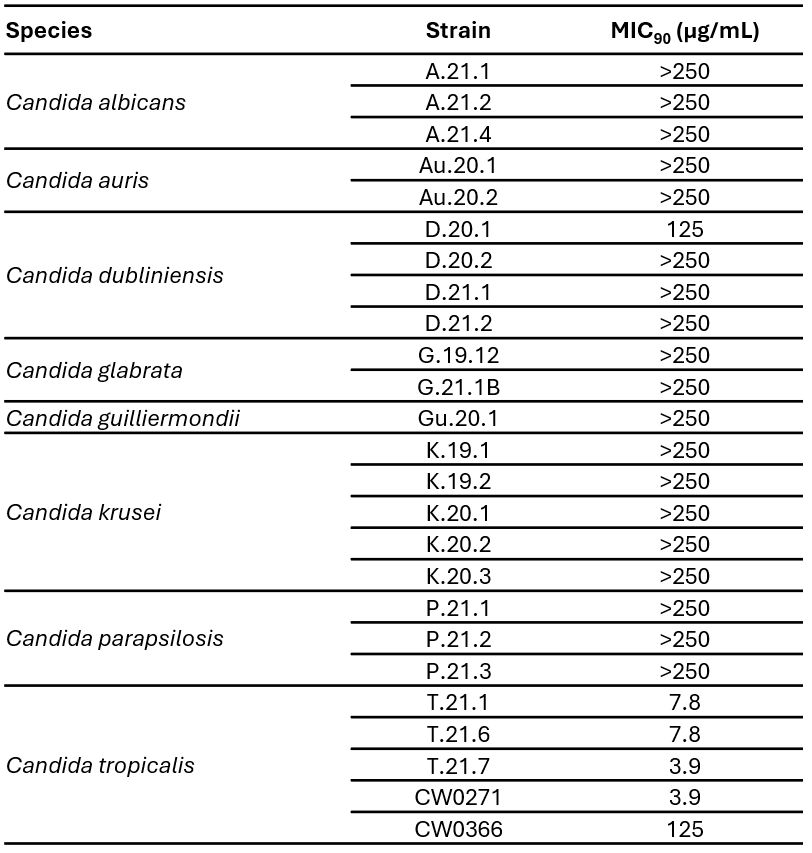


**^1^H and ^13^C NMR spectra of Compound 1**

**^
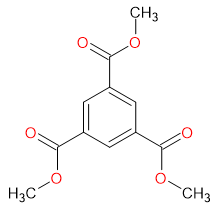
^**
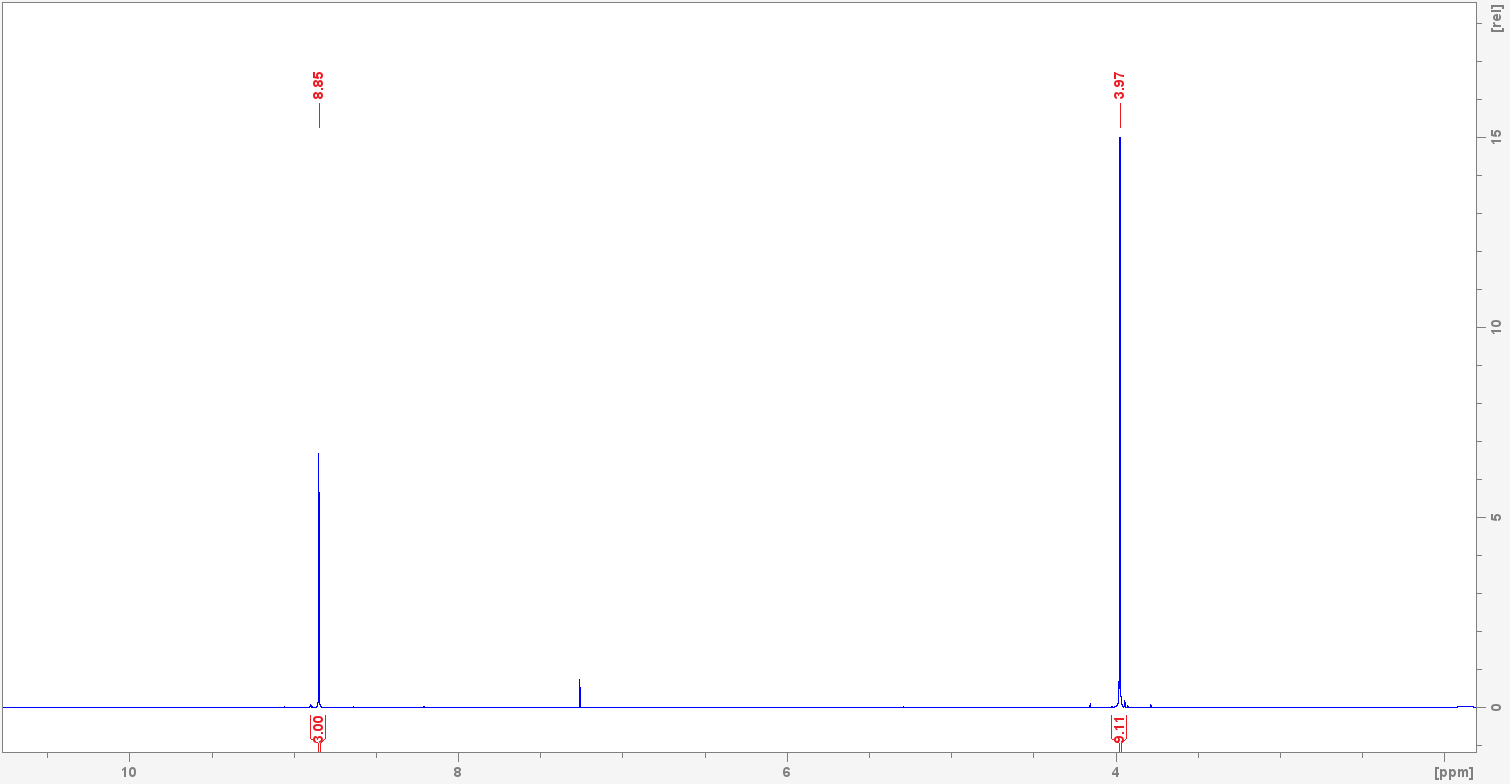


**^
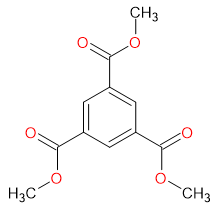
^**
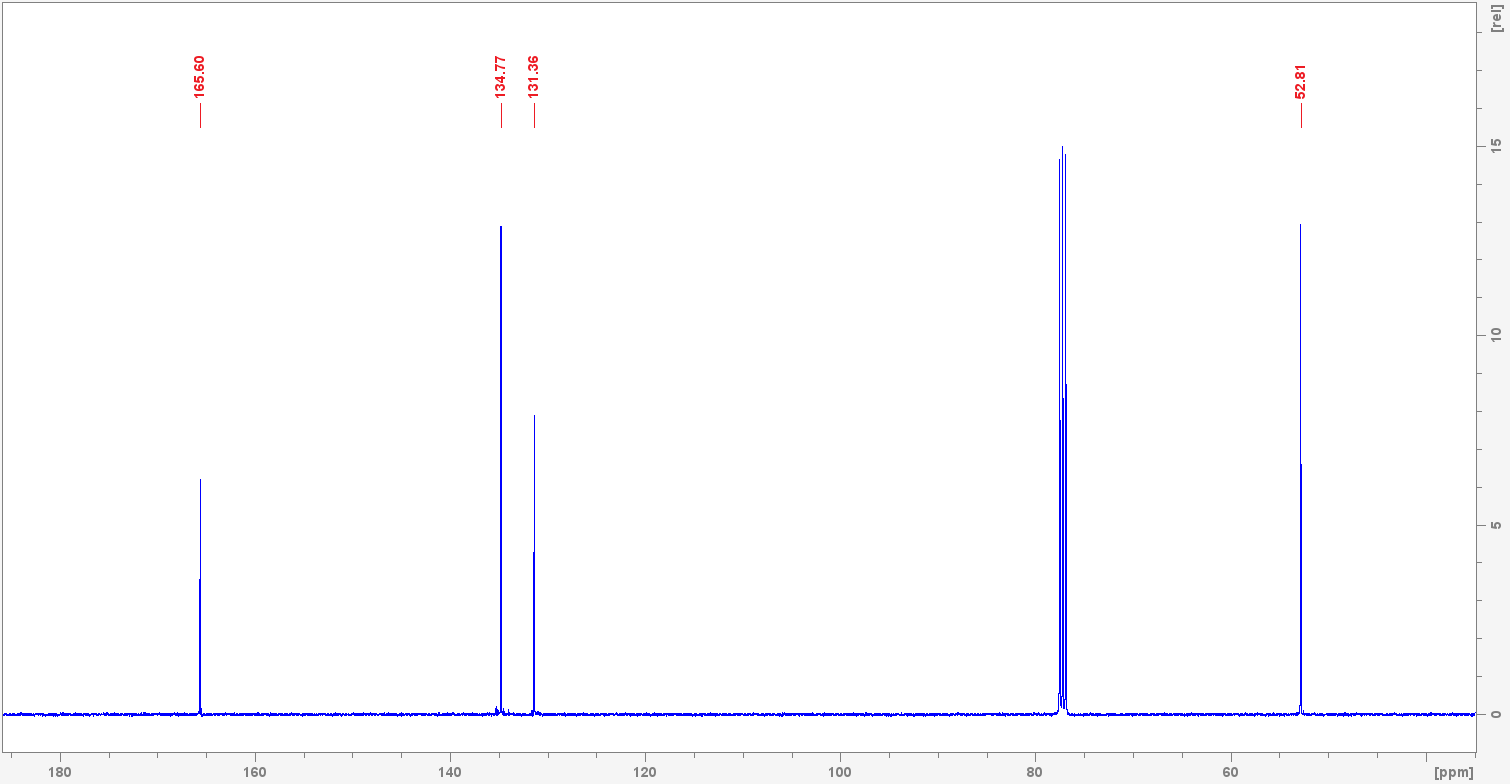


**^1^H and ^13^C NMR spectra of Compound 2**


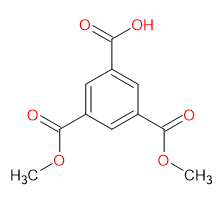

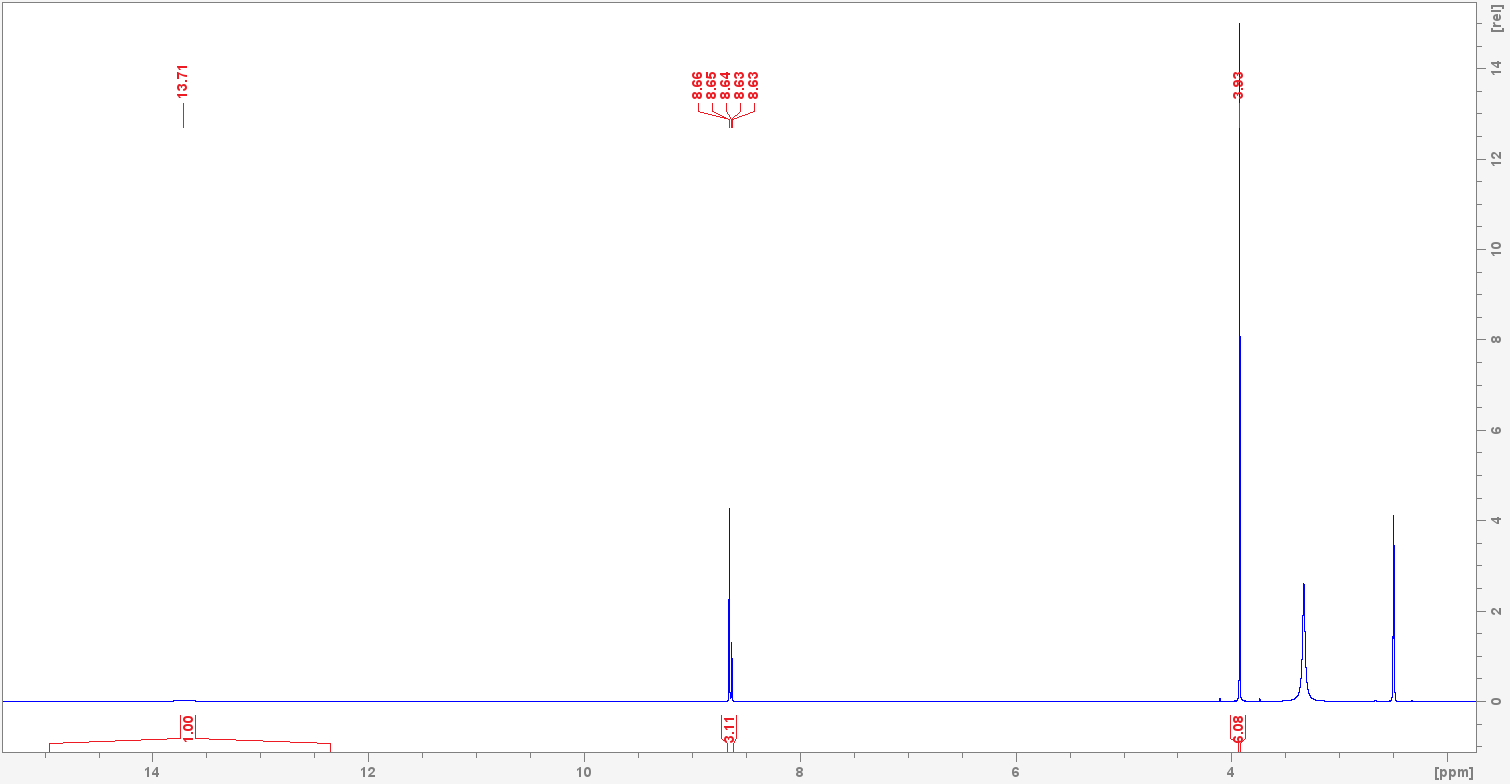


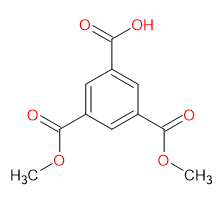

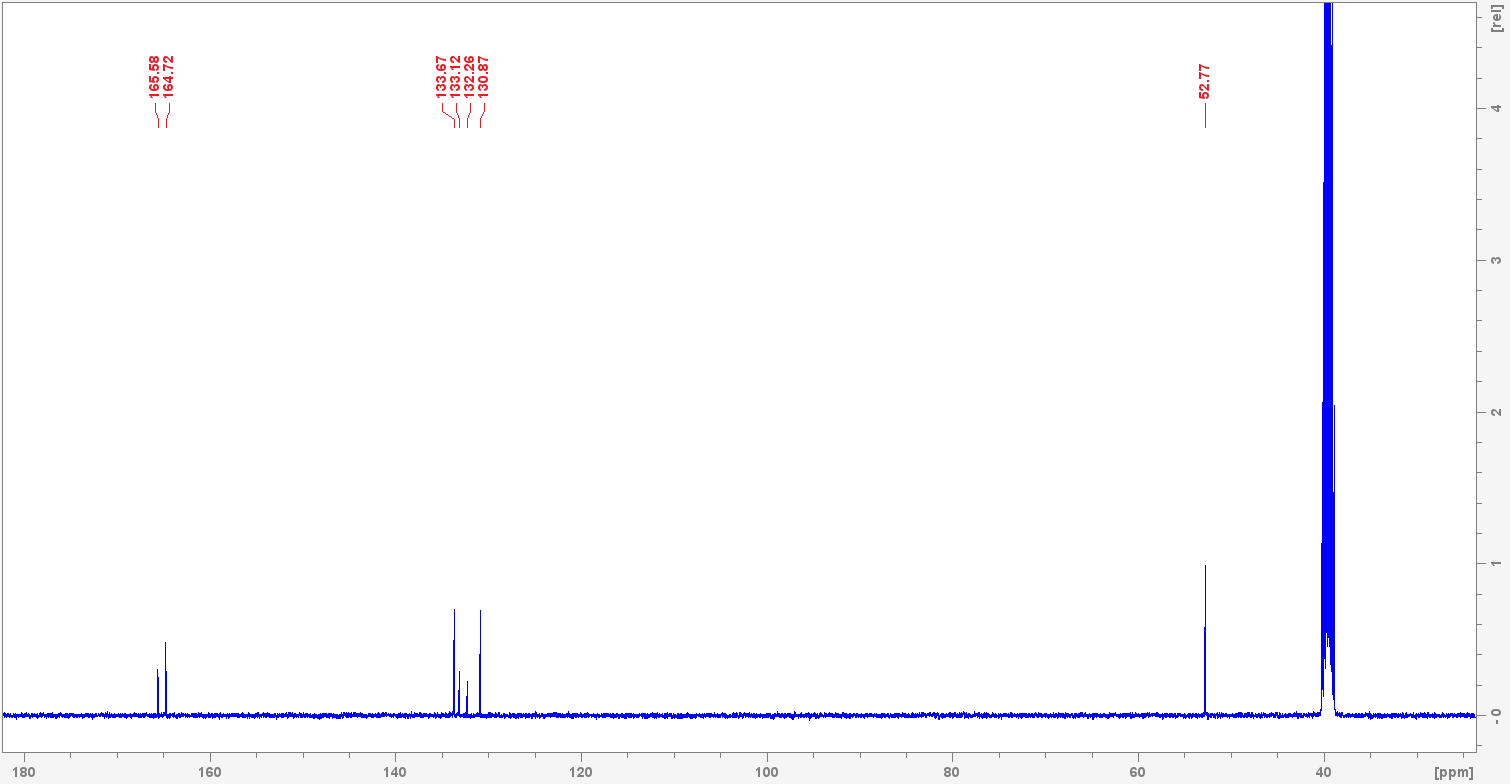


**^1^H and ^13^C NMR spectra of Compound 3**

**
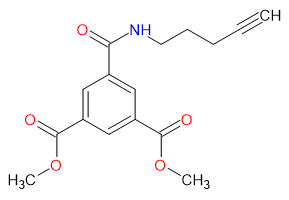
**
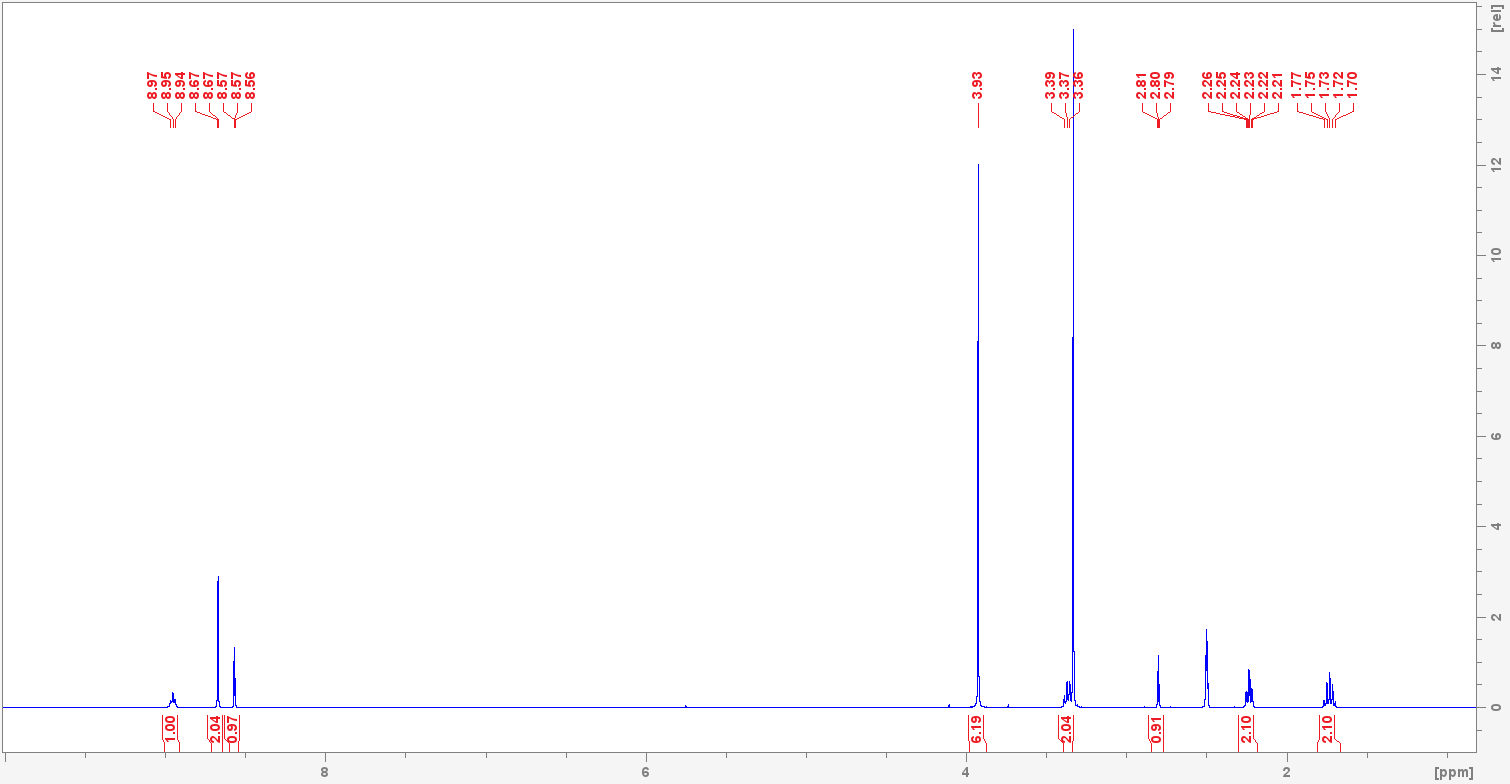


**
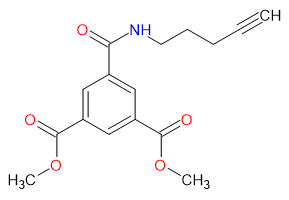
**
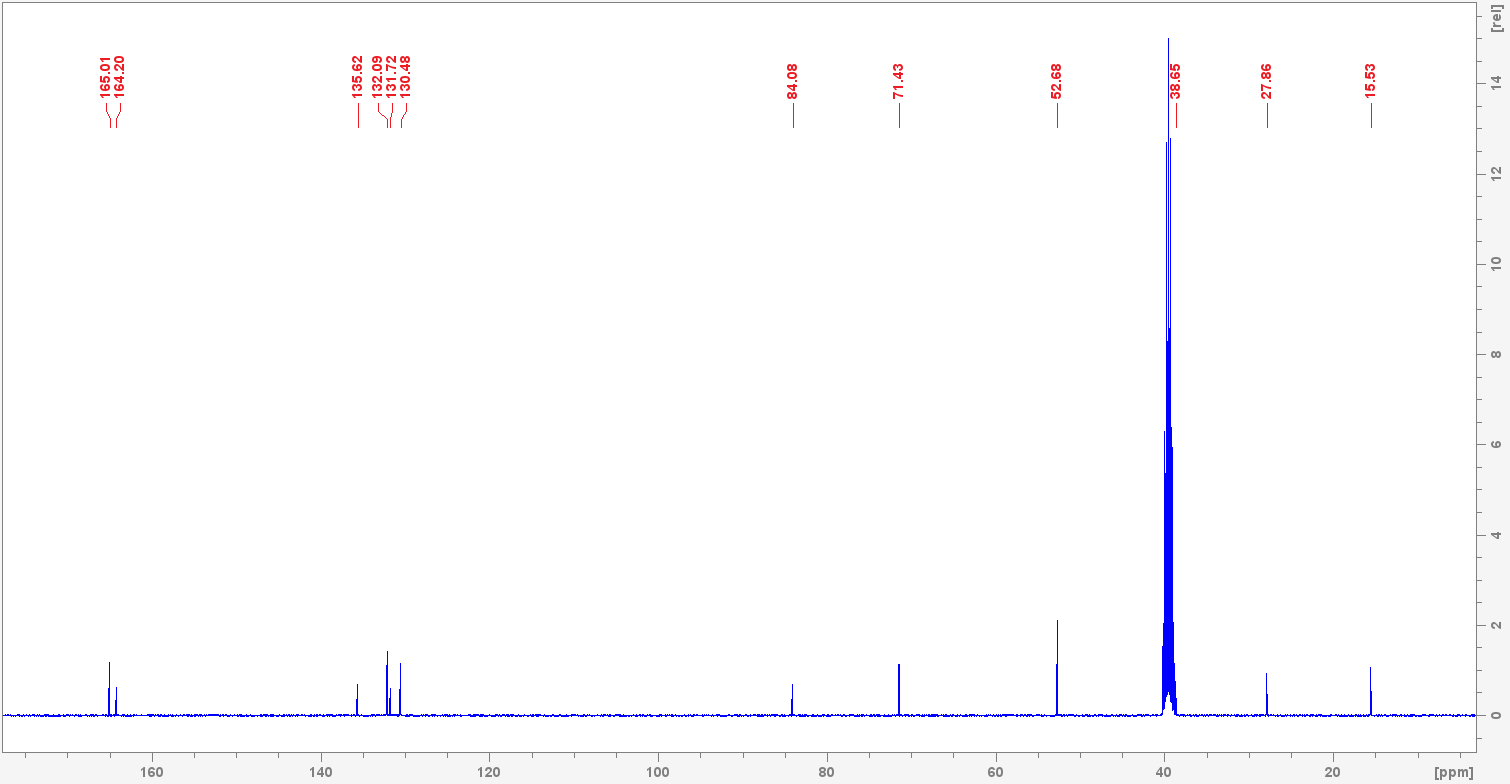


**^1^H and ^13^C NMR spectra of Compound 4**

**
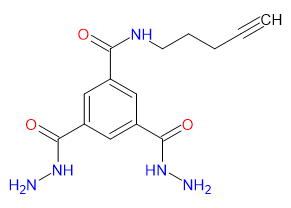
**
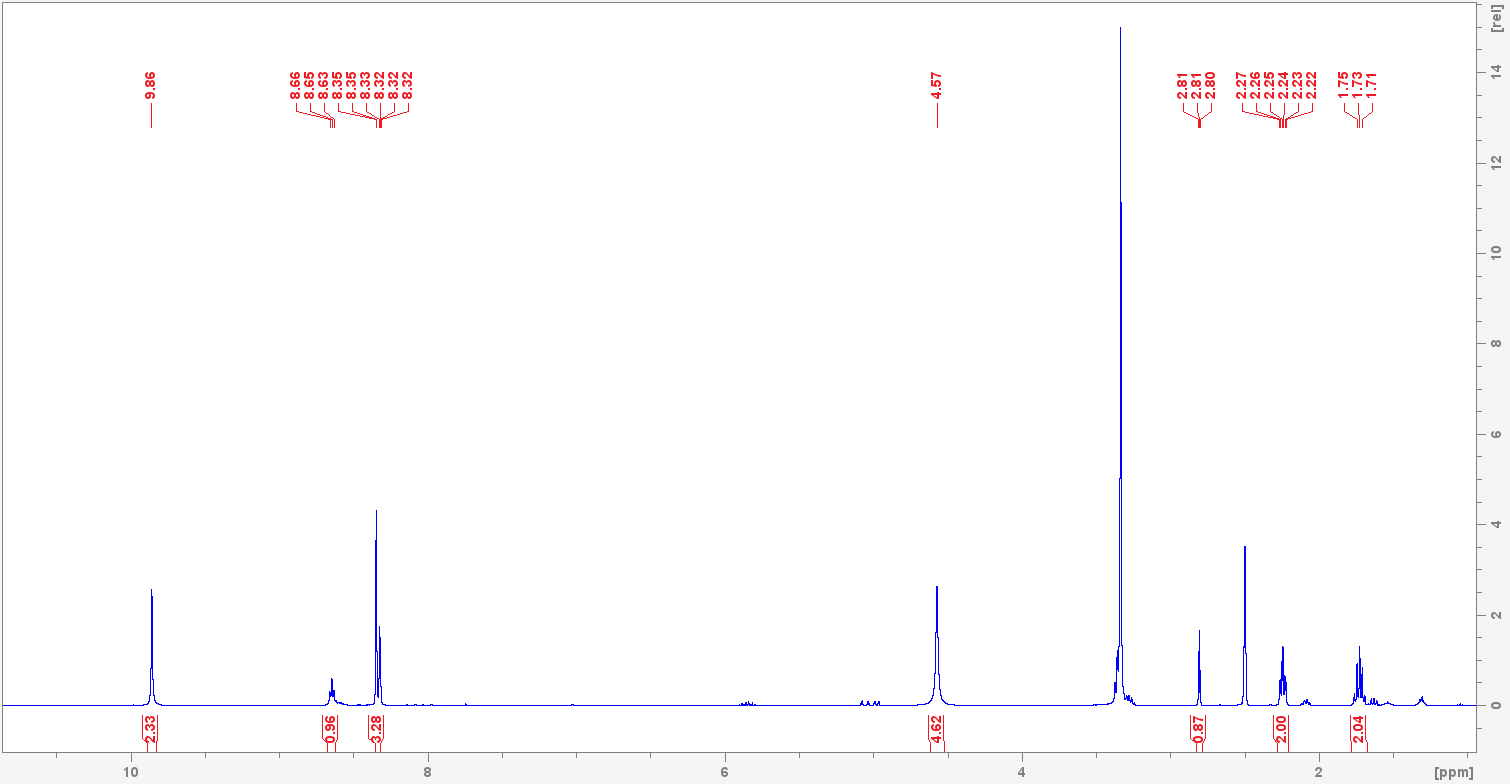


**
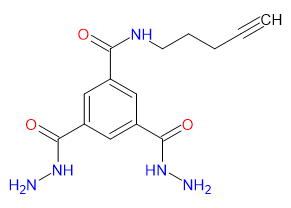
**
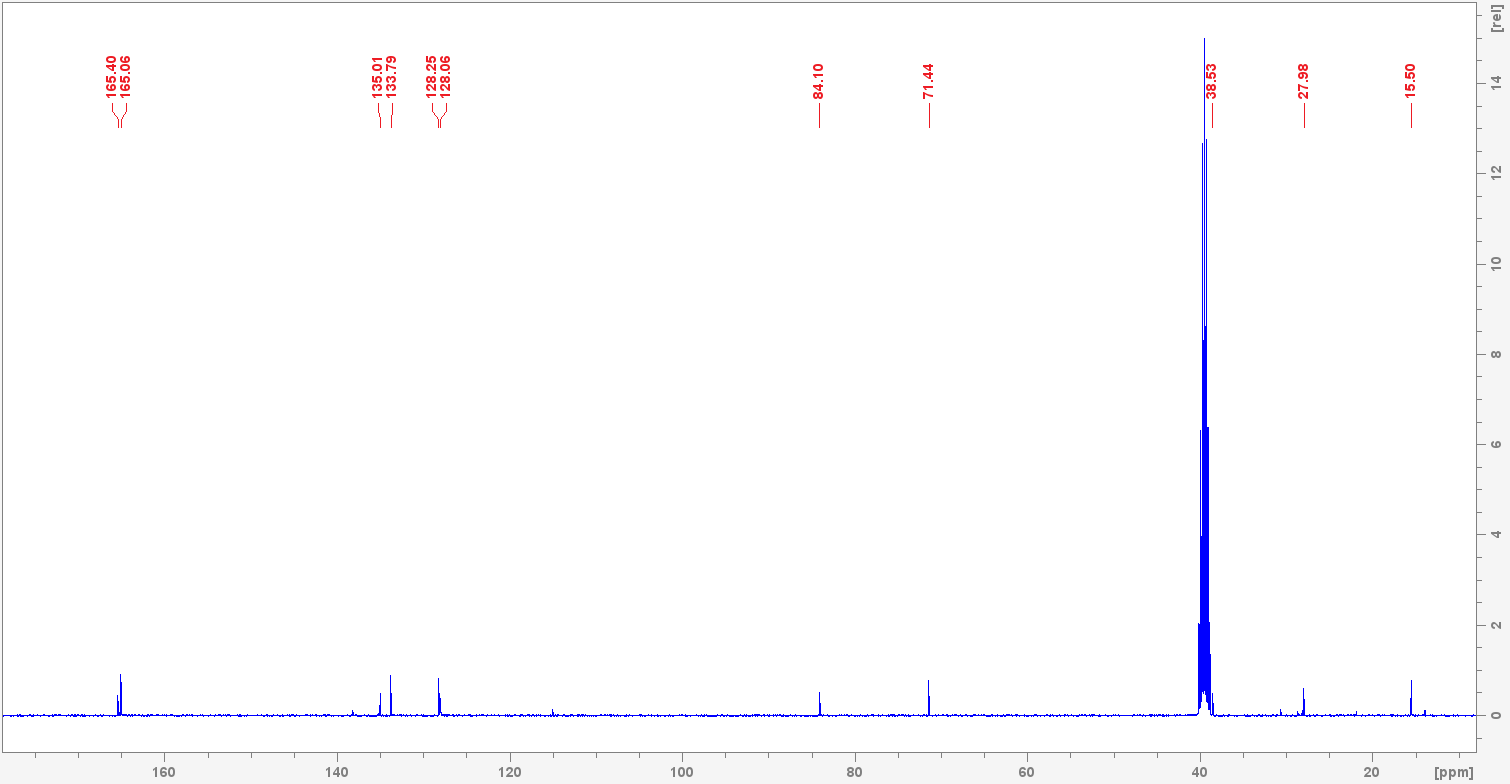


**^1^H and ^13^C NMR spectra of Compound 5**

**
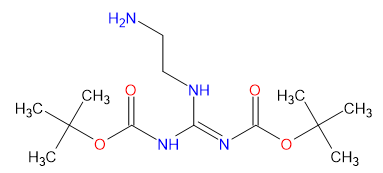
**
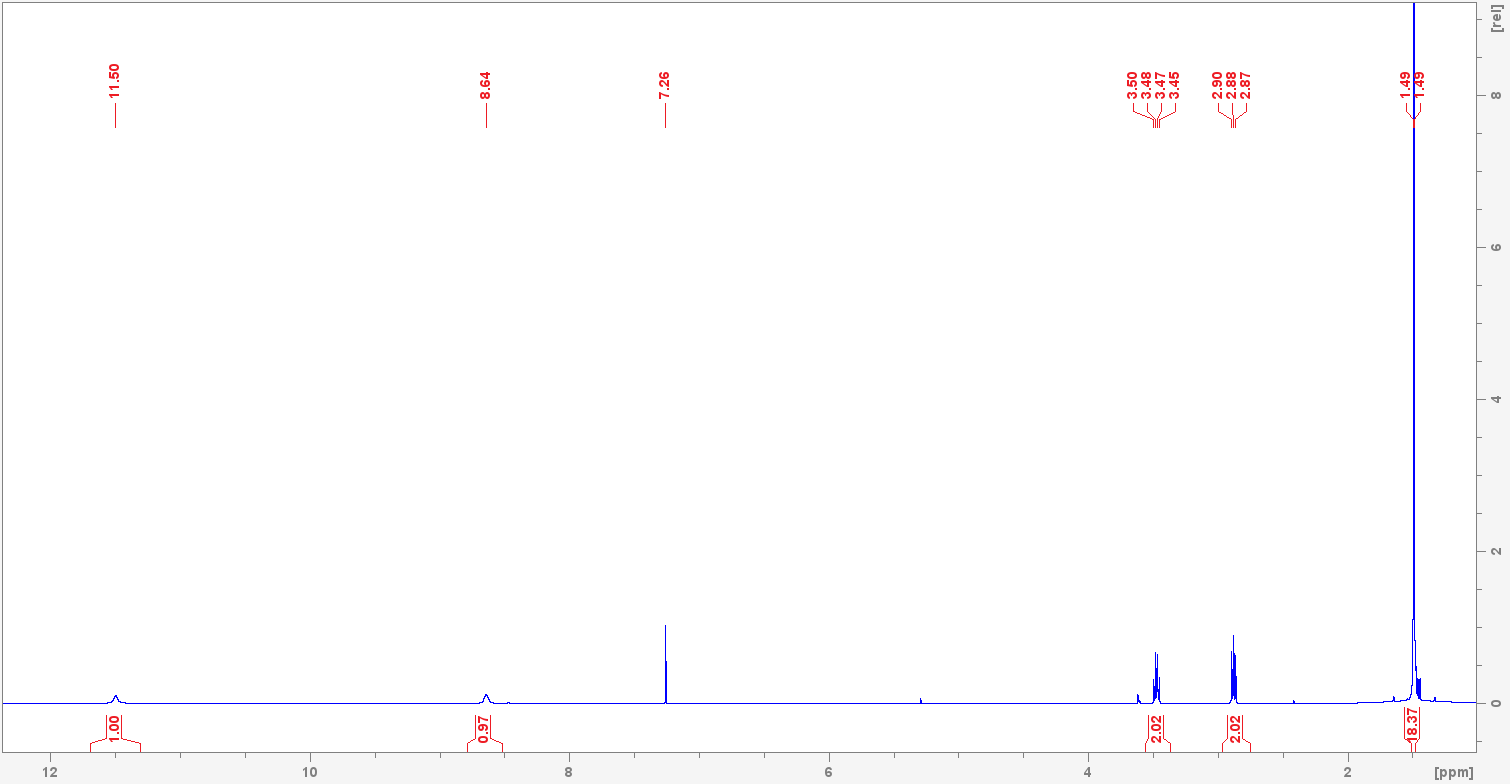


**
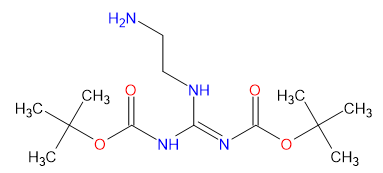
**
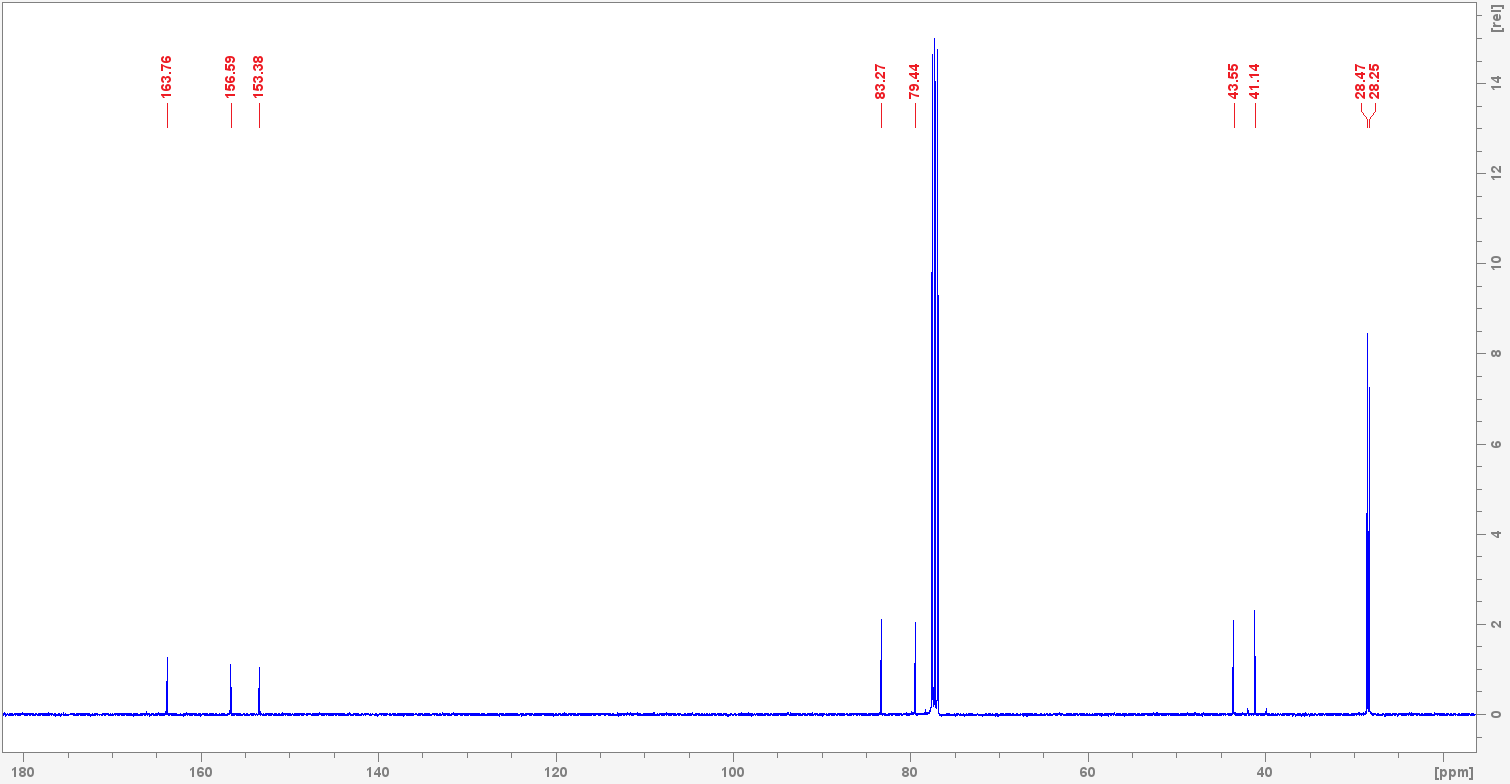


**^1^H and ^13^C NMR spectra of Compound 6**

**
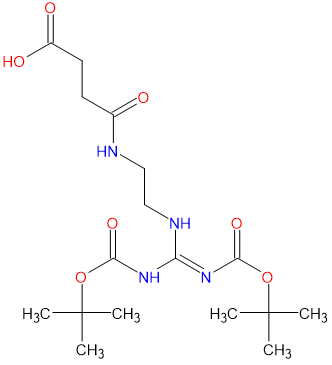
**
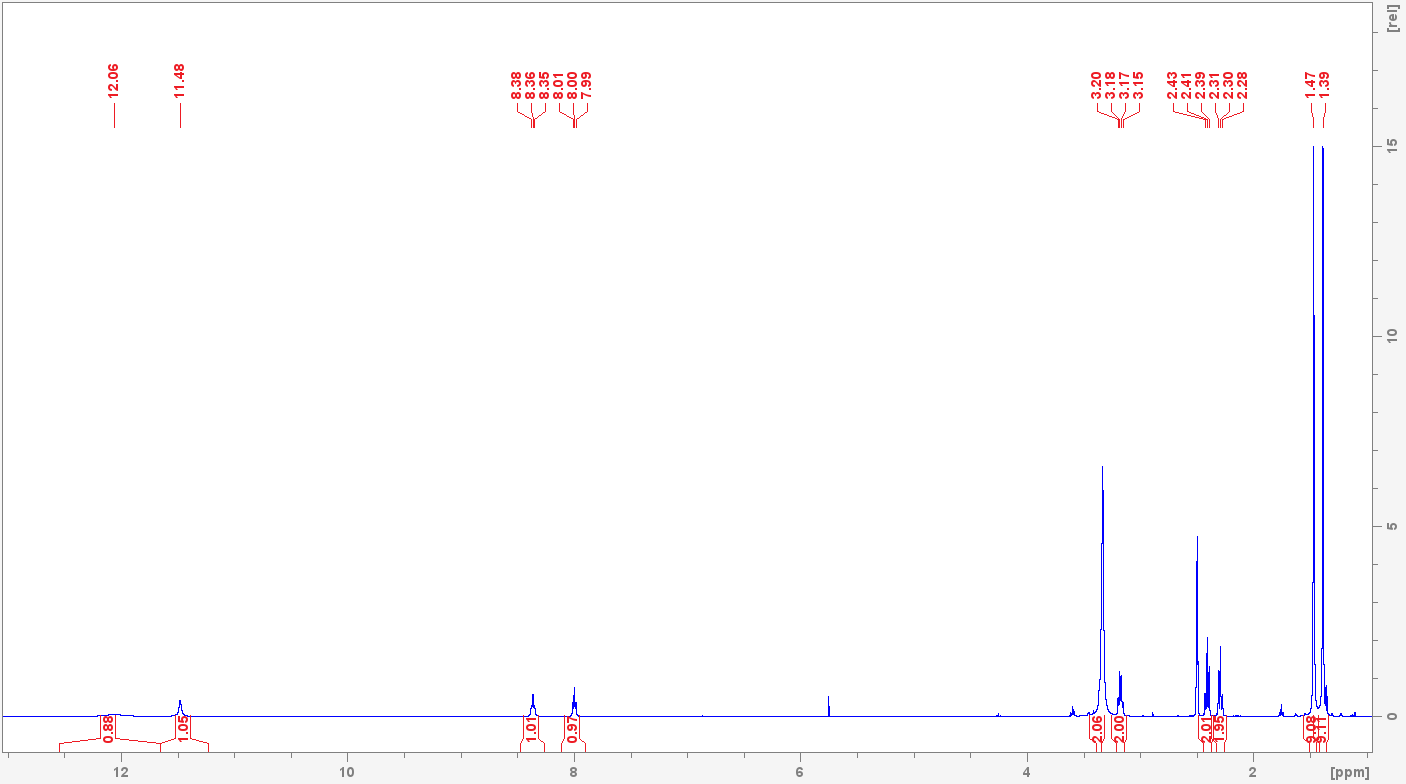


**
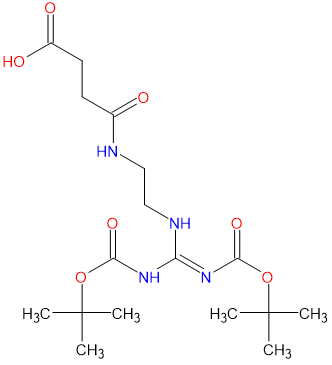
**
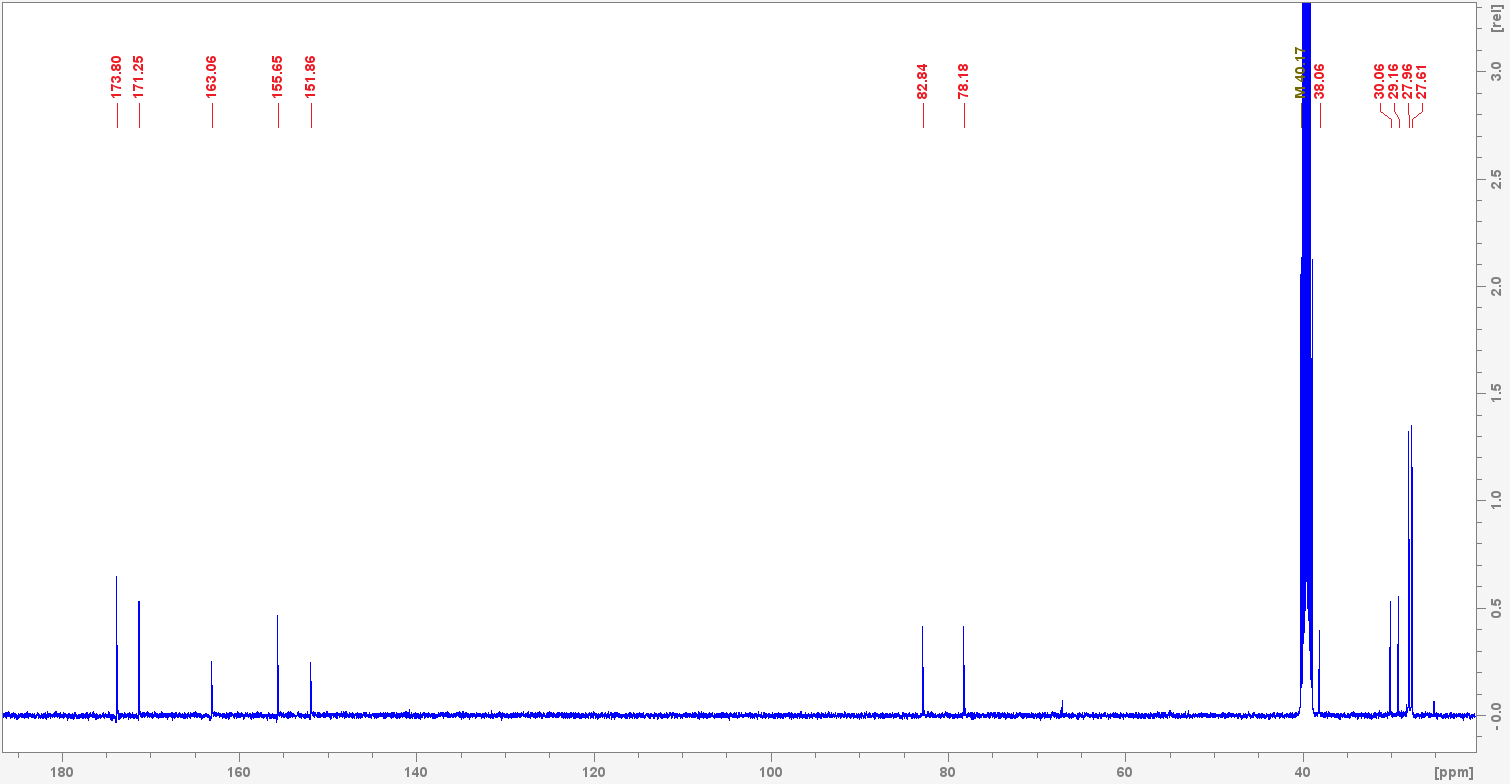


**^1^H and ^13^C NMR spectra of Compound 7**

**
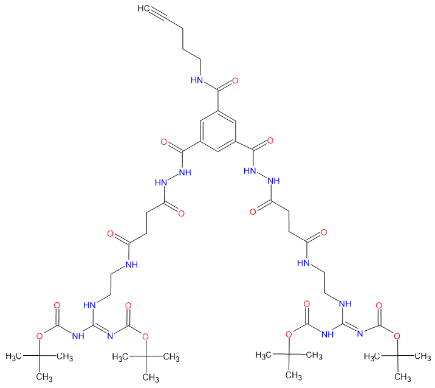
**
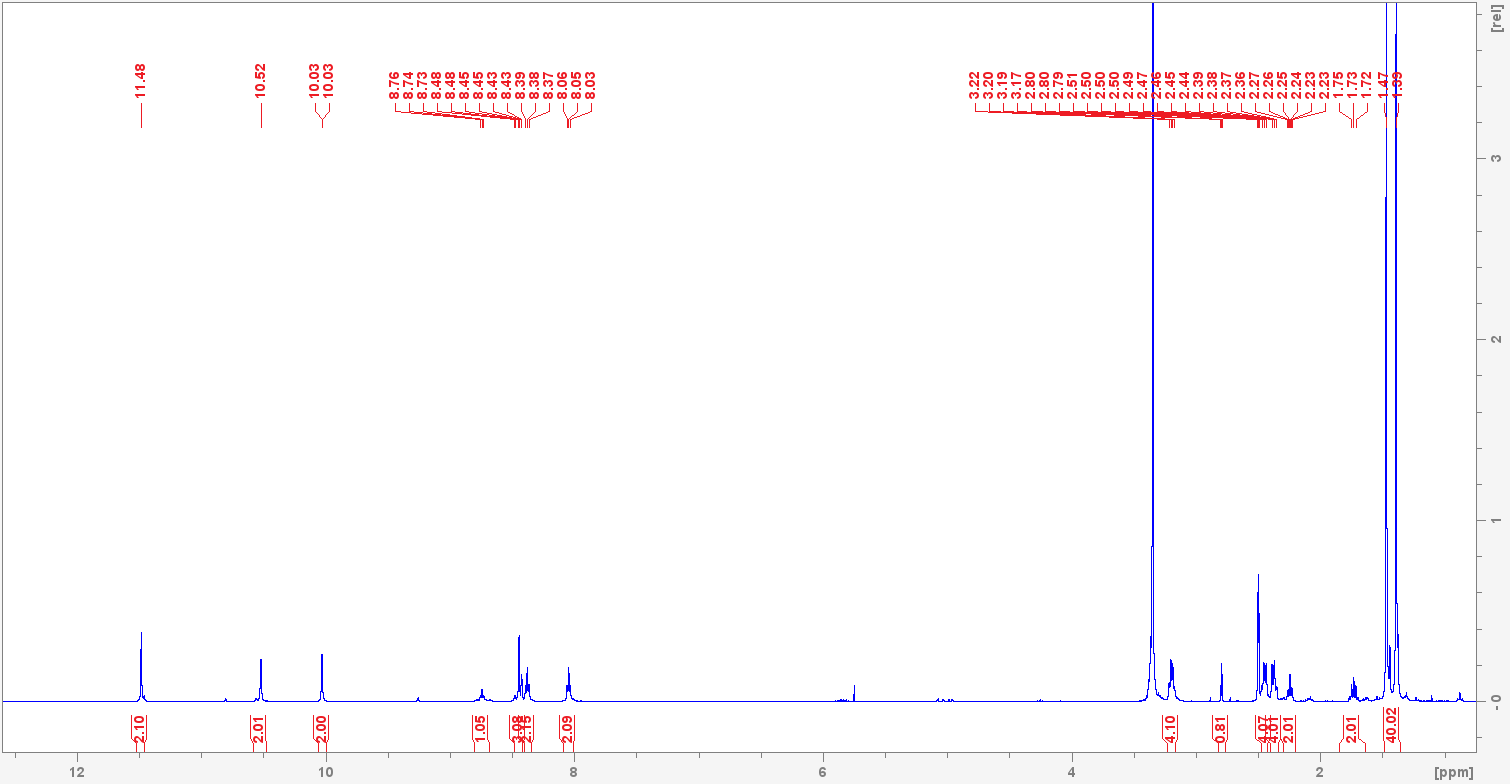


**
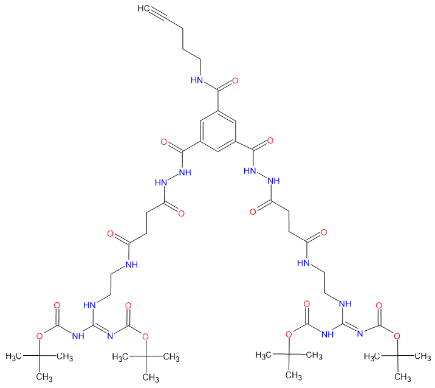
**
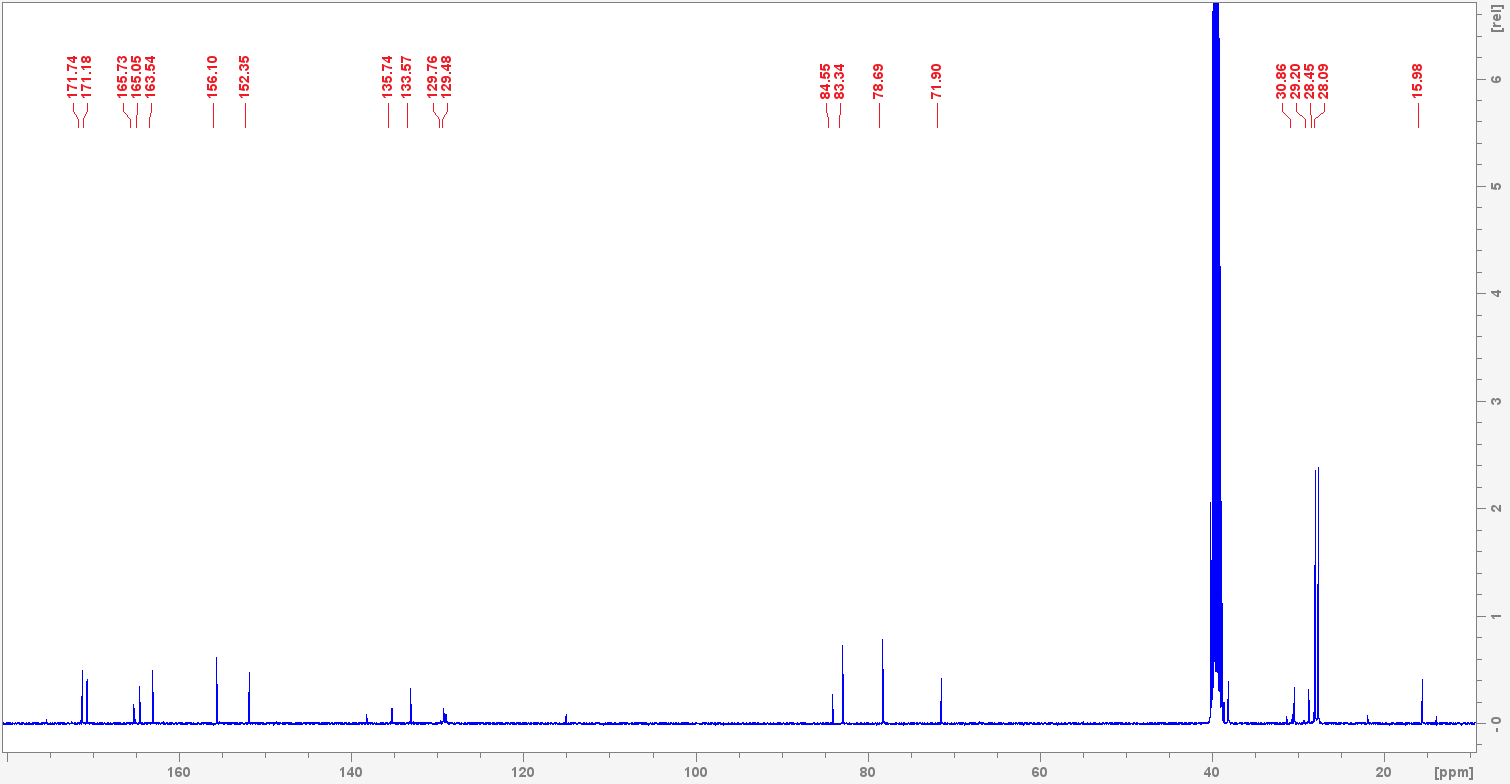


**^1^H NMR spectrum of Compound 9**


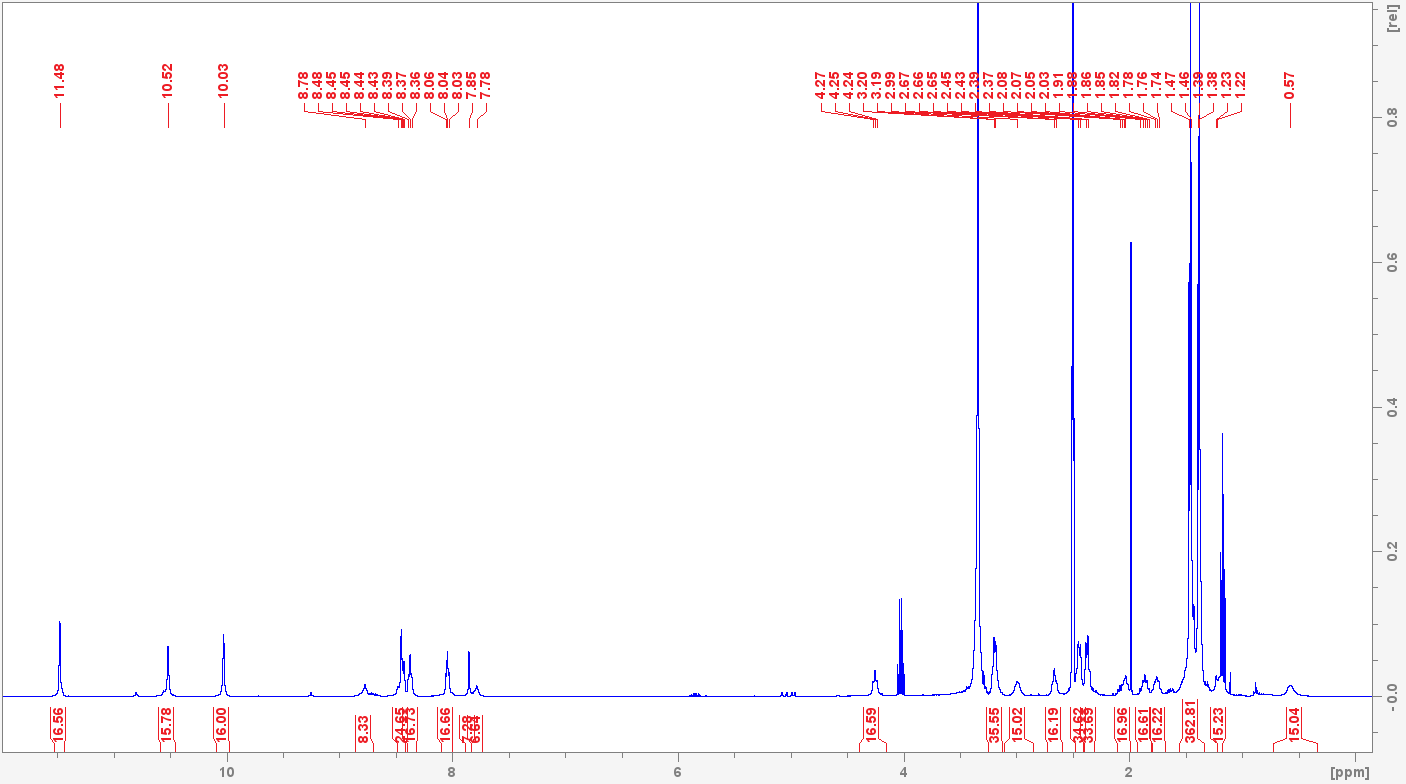


**^1^H NMR spectrum of Compound 10 (Gua-SMACS-16)**


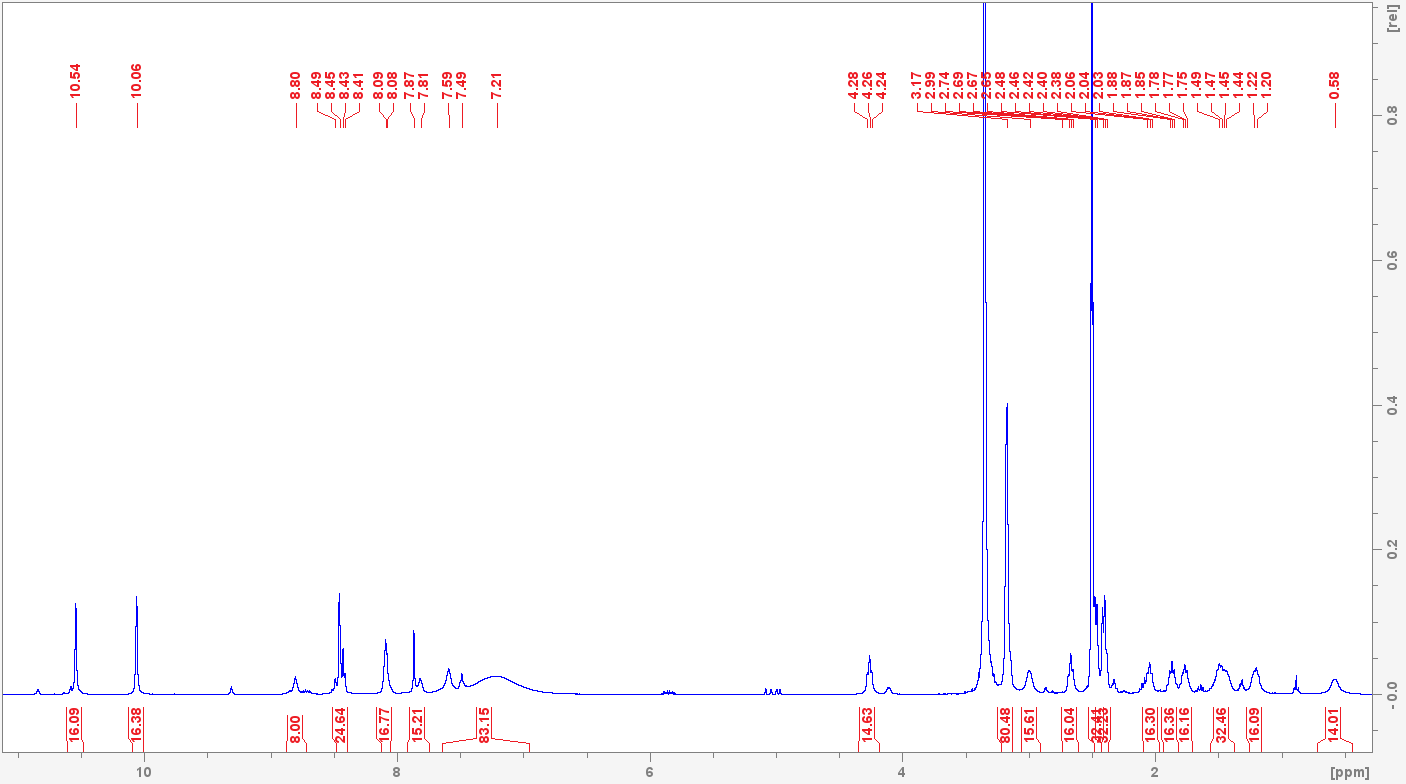


**^29^Si NMR spectrum of Compound 10 (Gua-SMACS-16)**


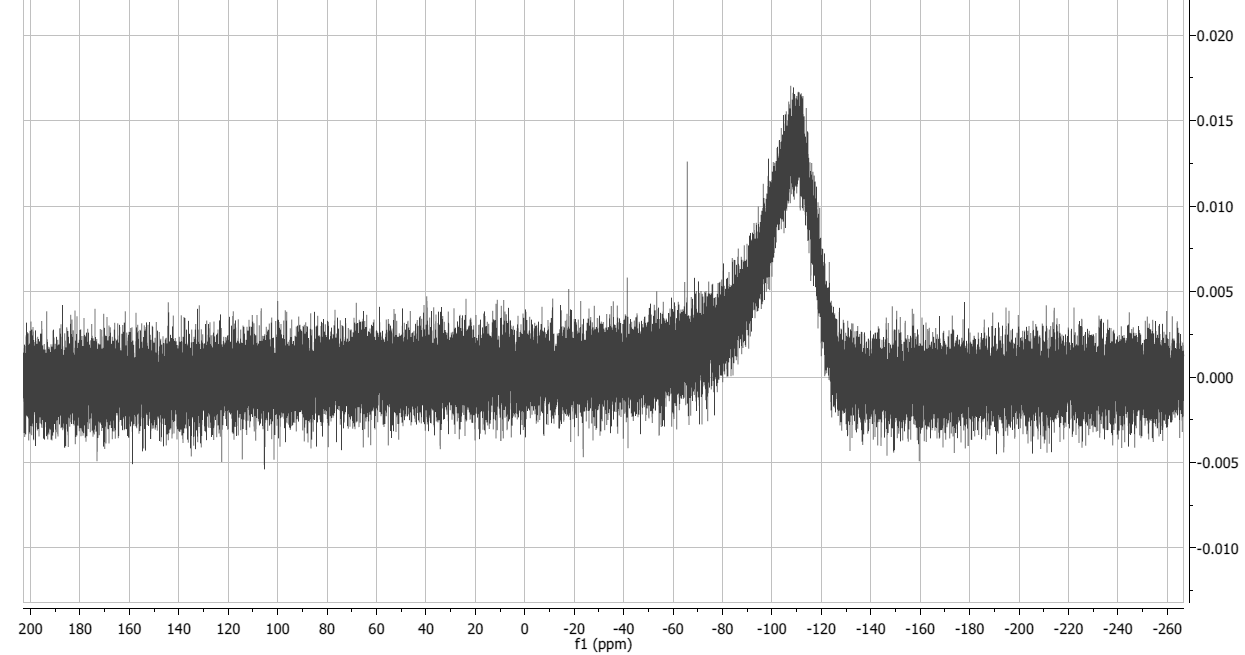


**LC-MS results for Compound 10 (Gua-SMACS-16)**


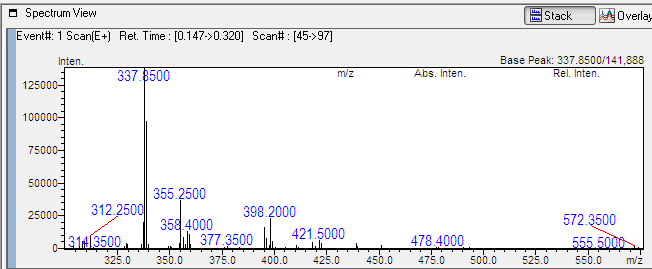


**GPC trace for Compound 10 (Gua-SMACS-16)**

**HPLC profile for Compound 10 (Gua-SMACS-16)**

**MALDI-TOF profile for Compound 10 (Gua-SMACS-16)**

**References**

[1] V. Yarlagadda, P. Akkapeddi, G. B. Manjunath, J. Haldar, *J. Med. Chem.* **2014**, *57*, 4558-4568.

[2] R. Lakshminarayanan, S. Liu, J. Li, M. Nandhakumar, T. T. Aung, E. Goh, J. Y. T. Chang, P. Saraswathi, C. Tang, S. R. B. Safie, L. Y. Lin, H. Riezman, Z. Lei, C. S. Verma, R. W. Beuerman, *PLOS ONE* **2014**, *9*, e87730.

[3] A. Kumar, H. Mohanram, J. Li, H. Le Ferrand, C. S. Verma, A. Miserez, *Chem. Mater.* **2020**, *32*, 8845-8859.

[4] F. S. Emami, V. Puddu, R. J. Berry, V. Varshney, S. V. Patwardhan, C. C. Perry, H. Heinz, *Chem. Mater.* **2014**, *26*, 2647-2658.

[5] D. A. Case, H. M. Aktulga, K. Belfon, D. S. Cerutti, G. A. Cisneros, V. W. D. Cruzeiro, N. Forouzesh, T. J. Giese, A. W. Götz, H. Gohlke, S. Izadi, K. Kasavajhala, M. C. Kaymak, E. King, T. Kurtzman, T.-S. Lee, P. Li, J. Liu, T. Luchko, R. Luo, M. Manathunga, M. R. Machado, H. M. Nguyen, K. A. O’Hearn, A. V. Onufriev, F. Pan, S. Pantano, R. Qi, A. Rahnamoun, A. Risheh, S. Schott-Verdugo, A. Shajan, J. Swails, J. Wang, H. Wei, X. Wu, Y. Wu, S. Zhang, S. Zhao, Q. Zhu, T. E. Cheatham, III, D. R. Roe, A. Roitberg, C. Simmerling, D. M. York, M. C. Nagan, K. M. Merz, Jr., *J. Chem. Inf. Model.* **2023**, *63*, 6183-6191.

[6] U. Essmann, L. Perera, M. L. Berkowitz, T. Darden, H. Lee, L. G. Pedersen, *J. Chem. Phys.* **1995**, *103*, 8577-8593.

[7] G. Bussi, D. Donadio, M. Parrinello, *J. Chem. Phys.* **2007**, *126*, 014101.

[8] M. Parrinello, A. Rahman, *J. Appl. Phys.* **1981**, *52*, 7182-7190.

[9] M. J. Abraham, T. Murtola, R. Schulz, S. Páll, J. C. Smith, B. Hess, E. Lindahl, *SoftwareX* **2015**, *1-2*, 19-25.

[10] N. Li, H.-K. Luo, A. X. Chen, J. P. K. Tan, C. Yang, M. J. Y. Ang, H. Zeng, Y. Y. Yang, *ACS Appl. Mater. Interfaces* **2023**, *15*, 354-363.
